# Supplementary material for: Mathematical modeling of plus-strand RNA virus replication to identify broad-spectrum antiviral treatment strategies
Source: PLoS Comput Biol. 2023 Apr 4;19(4):e1010423. doi: 10.1371/journal.pcbi.1010423 (PMC10104377; doi:10.1371/journal.pcbi.1010423)
Supplement: S1 Text — (DOCX) [file pcbi.1010423.s001.docx]

# S1 Text: Pan-viral and virus-specific parameters.

The hepatitis C virus (HCV) is one of the best-studied viruses, thus, several model parameters could be taken directly from the literature (Table 2). For dengue virus (DENV) and coxsackievirus B3 (CVB3), fewer replication process parameters and viral genome/protein half-lives have been measured experimentally. We used such experimentally determined parameters if available or alternatively used parameters experimentally determined from closely related plus-strand RNA viruses, i.e., Zika (ZIKV) and poliovirus (PV), respectively. DENV and ZIKV belong to the family *Flaviviridae* with genus *Flavivirus* and are both mosquito-borne plus-strand RNA viruses transmitted by the same *Aedes* species (*Aedes aegypti* and *Aedes albopictus*). Both virus genomes have a similar size (~11 kb) and organization, encoding seven non-structural (NS1, NS2A, NS2B, NS3, NS4A, NS4B, and NS5) and three structural proteins (C, prM/M, E) [1,2]. Furthermore, DENV and ZIKV both carry out their viral genome replication within invaginated replication organelles of the endoplasmic reticulum [3]. CVB3 and PV are also closely related plus-strand RNA viruses of the family *Picornaviridae* and genus *Enterovirus* with similar genome size (~7.5 kb) and organization; four structural proteins (VP1/2/3/4) and seven non-structural proteins (2A, 2B, 2C, 3A, 3B, 3C, and 3D). Most importantly, CVB3 and PV have a similar duration of their life cycles from virus entry to cell-lysis and release of thousands of progeny virions after around 8 hours [4].

Based on the virus-specific genome size (9.6 kb for HCV vs. 10.7 kb for DENV vs. 7.5 kb for CVB3, Table 1), we calculated the RNA translation rate $k_{2}^{i}$ as well as the virus RNA synthesis rates $k_{4m}^{i}=k_{4p}^{i}$ or took them from the literature. Calculations of the virus-specific RNA translation rates $k_{2}^{i}$ were based on the assumption that 3 to 8 amino acids per second are translated per polysome, which consists of 10 ribosomes [5]. For HCV, it has been shown that at least eight ribosomes are bound to an HCV subgenomic replicon that does not code for structural proteins [6]. Hence, we calculated the number of bound ribosomes and the viral RNA translation rate for full-length HCV (9.6 kb) and DENV genomes (10.7 kb), resulting in polysomes of 15 and 17 ribosomes for HCV and DENV, respectively. Considering polyprotein lengths of 3,200 and 3,400 amino acids for HCV and DENV, respectively, we calculated the viral RNA translation rates as $k_{2}^{i}\in\{HCV=180 h^{-1}, DENV= 100 h^{-1}\}$. Note that even though the RNA translation mechanism of HCV and DENV are different, where HCV (similar to CVB3) possesses an internal ribosome entry site (IRES) while DENV has a 5’ cap structure [7,8], we assume the same ribosome density for both viruses. For PV – as a representative of CVB3 – an RNA translation duration of 6.25 minutes per PV genome has been measured [9]. Further, it has been found that 35 to 40 ribosomes are initially bound to each PV RNA genome, whereas later during infection only 20 ribosomes are bound [9]. For simplicity, we assume a polysome complex consisting of 30 ribosomes per PV RNA genome and, thus, we calculated a translation rate of $k_{2}^{CVB3}=300 h^{-1}$.

Similarly, we calculated the virus-specific minus- and plus-strand synthesis rates ($k_{4m}^{i}=k_{4p}^{i}$). Here, we assume a synthesis rate of 180 nucleotides per minute [5]. By applying this RNA synthesis rate to the full-length genomes of HCV (9.6 kb) and DENV (10.7 kb), we obtained DENV $k_{4m}^{HCV}=k_{4p}^{HCV}=1.1 h^{-1}$ and $k_{4m}^{DENV}=k_{4p}^{DENV}=1.0 h^{-1}$ for HCV and DENV, respectively. The time needed to synthesize a whole PV genome is substantially shorter than for the *Flavivididae* representatives HCV and DENV and has been measured as 45 to 100 seconds (75 to 167 nucleotides per second), leading to a minus- and plus-strand synthesis rate of $k_{4m}^{CVB3}=k_{4p}^{CVB3}=50 h^{-1}$ [9].

We modeled the virus assembly and release ($v_{p}$, Eq. 15) similarly to our previously published DENV replication model [10]. We approximated the average virus concentration, for which enough structural proteins have to be available, from our experimental measurements as $K_{D}^{HCV}=0.04$ virions/mL, $K_{D}^{DENV}=1.8$ virions/mL, and $K_{D}^{CVB3}=40$ virions/mL. Note that we did not model virus assembly in detail. However, we account for the considerable number of structural proteins necessary for one virus particle by choosing the protein species with the highest consumption in the assembly process. In the case of CVB3, 60 copies of each structural protein (V1/2/3/4) are incorporated into one CVB3 ($N_{P_{S}}^{CVB3}=60$ structural proteins per virion) [11]. The envelope of DENV consists of an unspecified number of capsid (C) proteins as well as 180 copies of envelope (E) and 180 copies of membrane (prM) proteins. We assume that the number of C proteins does not exceed the numbers of E and prM, resulting in 180 structural proteins per DENV ($N_{P_{S}}^{DENV}=180$ structural proteins per virion) [12]. For HCV, the number of structural proteins is based on Aunins et al. (2018), who estimated the HCV core-to-RNA ratio with 180:1 ($N_{P_{S}}^{HCV}=180$ structural proteins per virion) [13].

Degradation rates of model species are based either on experimental half-lives or model-fitting results. For free cytosolic viral RNA, we found closely related degradation rates with $\mu_{R_{P}}^{i}\in\left\{ HCV=0.26 h^{-1}, DENV=0.23 h^{-1}, CVB3=PV=0.15 h^{-1} \right\}$ [13–15]. Similarly to our previously published HCV and DENV models [10,16], we assume that the translation complex $TC$ is more stable than free cytosolic viral RNA, $R_{P}$, due to the bound ribosomes, and thus we set $\mu_{TC}^{i}=0.5\cdot\mu_{RP}^{i}$. We took the degradation rate of viral RNA and protein species within the replication organelle from our previously published HCV model with $\mu_{RO}=0.086 h^{-1}$ as well as the degradation rate of the viral RNA translation marker luciferase with $\mu_{L}=0.35 h^{-1}$ [16]. We further assume that the degradation rate of species within the RO is lower than those of species in the cytoplasm and thus $\mu_{RO}^{i}\leq\mu_{RP}^{i}$, especially in the model that tests for the possibility of $\mu_{RC}^{i}$ being virus-specific (see S2 Text). Protein degradation rates have been experimentally measured and, thus, fixed to $\mu_{P}^{i}\in\{HCV=0.08 h^{-1}, DENV= 0.46 h^{-1}, CVB3=0.43 h^{-1}\}$ [15–17]. Similarly, the degradation rates of extracellular infectious virus have been assessed experimentally and were fixed to $\mu_{V}^{i}\in\left\{ HCV=0.1 h^{-1},DENV=ZIKV=0.13 h^{-1},CVB3=0.08 h^{-1} \right\}$ [18–21]. We set the degradation rate of intracellular virus within endosomes to $\mu_{V_{E}}=0.23 h^{-1}$ as it has been measured for ZIKV [12].

Note that since it is not clear how many viruses bind to the cell surface, the initial concentration for infectious virus was estimated for all three viruses, instead of fixing $V_{0}^{i}$ to the virus-specific MOI value. Nevertheless, since the MOI implies multiple rounds of infection, $k_{re}$ has been estimated. All parameter values are given in Table 2.

1. Suwanmanee S, Luplertlop N. Dengue and Zika viruses: lessons learned from the similarities between these Aedes mosquito-vectored arboviruses. Journal of Microbiology. Microbiological Society of Korea; 2017. pp. 81–89. doi:10.1007/s12275-017-6494-4

2. Sukhralia S, Verma M, Gopirajan S, Dhanaraj PS, Lal R, Mehla N, et al. From dengue to Zika: the wide spread of mosquito-borne arboviruses. European Journal of Clinical Microbiology and Infectious Diseases. Springer Verlag; 2019. pp. 3–14. doi:10.1007/s10096-018-3375-7

3. Cortese M, Goellner S, Acosta EG, Neufeldt CJ, Oleksiuk O, Lampe M, et al. Ultrastructural characterization of Zika virus replication factories. Cell Rep. 2017;18: 2113–2123. doi:10.1016/j.celrep.2017.02.014

4. Modrow S, Falke D, Truyen U, Schätzl H, Modrow S, Falke D, et al. Viruses with single-stranded, positive-sense RNA genomes. Molecular Virology. Springer Berlin Heidelberg; 2013. pp. 185–349. doi:10.1007/978-3-642-20718-1_14

5. Dahari H, Ribeiro RM, Rice CM, Perelson AS. Mathematical modeling of subgenomic hepatitis C virus replication in Huh-7 cells. J Virol. 2007;81: 750–60. doi:10.1128/JVI.01304-06

6. Wang C, Pflugheber J, Sumpter R, Sodora DL, Hui D, Sen GC, et al. Alpha interferon induces distinct translational control programs to suppress hepatitis C virus RNA replication. J Virol. 2003;77: 3898–3912. doi:10.1128/jvi.77.7.3898-3912.2003

7. Lukavsky PJ. Structure and function of HCV IRES domains. Virus Res. 2009;139: 166–171. doi:10.1016/j.virusres.2008.06.004

8. Gebhard LG, Filomatori C V., Gamarnik A V. Functional RNA elements in the dengue virus genome. Viruses. 2011;3: 1739–1756. doi:10.3390/v3091739

9. Regoes RR, Crotty S, Antia R, Tanaka MM. Optimal replication of poliovirus within cells. Am Nat. 2005;165: 364–73. doi:10.1086/428295

10. Zitzmann C, Schmid B, Ruggieri A, Perelson AS, Binder M, Bartenschlager R, et al. A coupled mathematical model of the intracellular replication of dengue virus and the host cell immune response to infection. Front Microbiol. 2020;11: 725. doi:10.3389/fmicb.2020.00725

11. Garmaroudi FS, Marchant D, Hendry R, Luo H, Yang D, Ye X, et al. Coxsackievirus B3 replication and pathogenesis. 2015;10: 629–652.

12. Persaud M, Martinez-Lopez A, Buffone C, Porcelli SA, Diaz-Griffero F. Infection by Zika viruses requires the transmembrane protein AXL, endocytosis and low pH. Virology. 2018;518: 301–312. doi:10.1016/j.virol.2018.03.009

13. Aunins TR, Marsh KA, Subramanya G, Uprichard SL, Perelson AS, Chatterjee A. Intracellular hepatitis C modeling predicts infection dynamics and viral protein mechanisms. J Virol. 2018;92: JVI.02098-17. doi:10.1128/JVI.02098-17

14. Simoes EA, Sarnow P. An RNA hairpin at the extreme 5’ end of the poliovirus RNA genome modulates viral translation in human cells. J Virol. 1991;65: 913–921. doi:10.1128/jvi.65.2.913-921.1991

15. Byk LA, Iglesias NG, De Maio FA, Gebhard LG, Rossi M, Gamarnik A V. Dengue virus genome uncoating requires ubiquitination. MBio. 2016;7: e00804-16. doi:10.1128/mBio.00804-16

16. Binder M, Sulaimanov N, Clausznitzer D, Schulze M, Hüber CM, Lenz SM, et al. Replication vesicles are load- and choke-points in the hepatitis C virus lifecycle. PLoS Pathog. 2013;9: e1003561. doi:10.1371/journal.ppat.1003561

17. Gohara DW, Arnold JJ, Cameron CE. Poliovirus RNA-dependent RNA polymerase (3Dpol): Kinetic, thermodynamic, and structural analysis of ribonucleotide selection. Biochemistry. 2004;43: 5149–5158. doi:10.1021/bi035429s

18. Zitzmann C, Kaderali L, Perelson AS. Mathematical modeling of hepatitis C RNA replication, exosome secretion and virus release. PLoS Comput Biol. 2020;16: e1008421. doi:10.1371/journal.pcbi.1008421

19. Carson SD, Chapman NM, Hafenstein S, Tracy S. Variations of coxsackievirus B3 capsid primary structure, ligands, and stability Are selected for in a coxsackievirus and adenovirus receptor-limited environment. J Virol. 2011;85: 3306–3314. doi:10.1128/jvi.01827-10

20. Carson SD, Hafenstein S, Lee H. MOPS and coxsackievirus B3 stability. Virology. 2017;501: 183–187. doi:10.1016/j.virol.2016.12.002

21. Goo L, Dowd KA, Smith ARY, Pelc RS, Demaso CR, Pierson TC. Zika virus is not uniquely stable at physiological temperatures compared to other flaviviruses. MBio. 2016;7. doi:10.1128/mBio.01396-16
