# Supplementary material for: Mathematical modeling of plus-strand RNA virus replication to identify broad-spectrum antiviral treatment strategies
Source: PLoS Comput Biol. 2023 Apr 4;19(4):e1010423. doi: 10.1371/journal.pcbi.1010423 (PMC10104377; doi:10.1371/journal.pcbi.1010423)
Supplement: S2 Text — (DOCX) [file pcbi.1010423.s002.docx]

# S2 Text: Model selection process.

Model selection was an iterative process of fitting the model (adding one virus-specific process at a time) to the experimental measurements of the three studied viruses – hepatitis C (HCV), dengue (DENV), and coxsackievirus B3 (CVB3). We considered the following 13 processes as possibly virus-specific:

Initial infection processes:

(1) receptor mediated virus entry $k_{e}$ and

(2) release of viral RNA $k_{f}$.

The only host factor we included in the model that is recruited by all plus-strand RNA viruses:

(3) the intracellular number of ribosomes available for viral RNA translation $Ribo_{tot}$.

RNA translation related processes in the cytoplasm:

(4) the formation of the translation complex $k_{1}$ and

(5) polyprotein cleavage $k_{c}$.

Processes related to the replication organelle (RO):

(6) the formation of the replicase complex $k_{Pin}$,

(7) the maximal concentration of replicase complexes within the RO $RC_{MAX}$,

(8) the formation of the double-strand intermediate complex $k_{5}$, and

(9) further replication within the RO $k_{3}$.

Processes following the successful viral synthesis:

(10) viral RNA export into the cytoplasm $k_{Pout}$ and

(11) virus assembly and release $k_{p}$.

Degradation rates for

(12) intracellular virus within endosomes $\mu_{VE}$ and

(13) viral RNA and protein species within the RO $\mu_{RO}$.

Note that we set processes as virus-specific if experimental measurements for all studied viruses were available. For more details to the mathematical model see Main text and S1 Text.

The iterative process of selecting models with virus-specific processes is illustrated in Fig A. Model implementation and fitting has been performed with the Data2Dynamics toolbox for the MATLAB [1,2]. The models have been fitted simultaneously to the virus-specific data sets, parameters have been estimated with a deterministic trust region algorithm (lsqnonlin) with Latin hyper cube multi-start by minimizing the log-likelihood function [2]:

$$\begin{aligned} \hat{L}=-2\log\left( L\left( \hat{y} | \theta\right) \right), \text{with } \\ L\left( \hat{y} | \theta\right)=\prod_{k=1}^{m} \prod_{i=1}^{d_{k}} \frac{1}{\sqrt{2\pi\sigma_{ki}^{2}}}\exp\left( -\frac{1}{2\sigma_{ki}^{2}}\left( \hat{y}_{ki}-y_{k}\left( t_{i},\theta\right) \right)^{2} \right),\#(S1) \\ \# \end{aligned}$$

implemented in Data2Dynamics [1].

Here, we minimize the difference between the observables ($y$) and the experimental data ($\hat{y}$) and a given set of model parameters ($\theta$); $m$ the number of observables ($k=1\ldots m$), $d_{k}$ the number of experimental data, $t_{i}$ the measurement time points ($i=1\ldots d_{k})$, and $\sigma_{ki}^{2}$ the variance of each experimental data point.

The Akaike information criterion (AIC) has been calculated with

$$\begin{aligned} AIC= \hat{L}+2K,\#\left( S2 \right) \end{aligned}$$

where $K$ is the number of model parameters. Note that we preferred the model with an AIC difference less than two ($\Delta AIC\leq2$), according to model selection theory [3].

Parameter identifiability has been performed by the profile likelihood estimation method implemented in the Data2Dynamics toolbox [4,5].


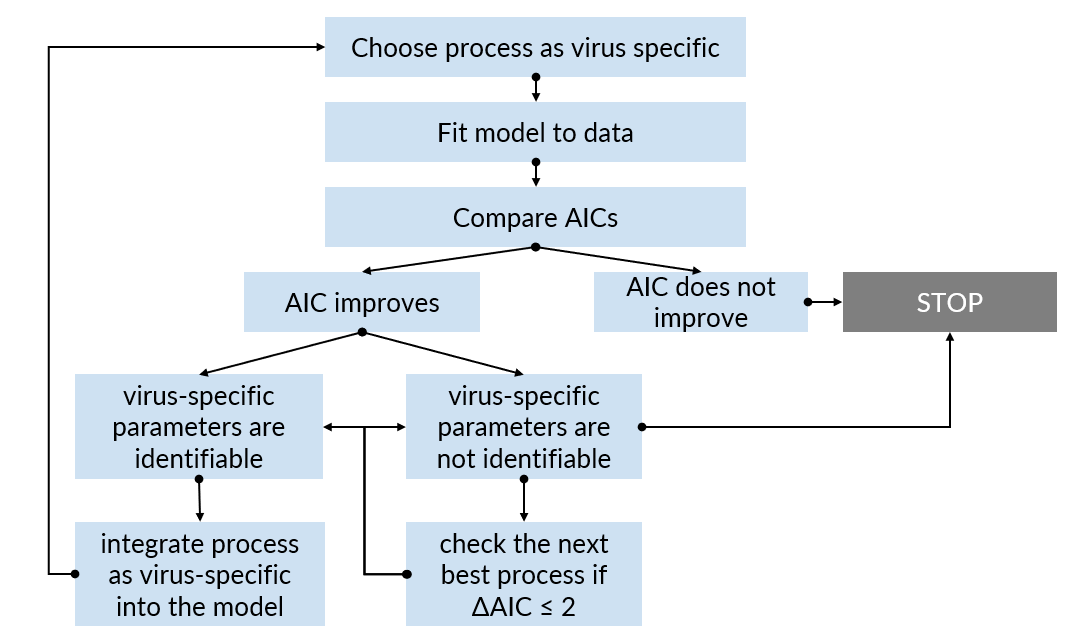


Fig A: Schematic illustration of the model selection process.

## 1. Basic model

As a starting point, we adapted our previously published models for HCV and DENV [6,7] by considering a maximal number of replicase complexes ($RC$) and, thus, multiplied the replicase complex formation process $k_{Pin}$ with $\left( 1-\frac{RC}{RC_{MAX}} \right)$. Although integrating another parameter into the model, we improved the AIC by around 700, compared to a model without a saturation in replicase complexes (see Table A). As shown in Figs B and D, the model with $RC_{MAX}$ captures the dynamics of the experimental measurements of all three viruses and improves the model fit*.* Furthermore, the model shows a high degree of identifiability where only two of the estimated parameters hit the upper parameter estimation bound (Figs C and E). Note that the maximal number of replicase complexes was not virus-specific and, thus, for now represented a pan-viral parameter.

Table A: Negative log likelihood (-LL), AIC and number of parameters (#p) for the model with and without $R_{RC_{MAX}}$.

|  | -LL | AIC | #p |
| --- | --- | --- | --- |
| Basic model without $RC_{MAX}$ | 2594 | 2684 | 45 |
| **Basic model with** $RC_{MAX}$ | **1890** | **1982** | **46** |


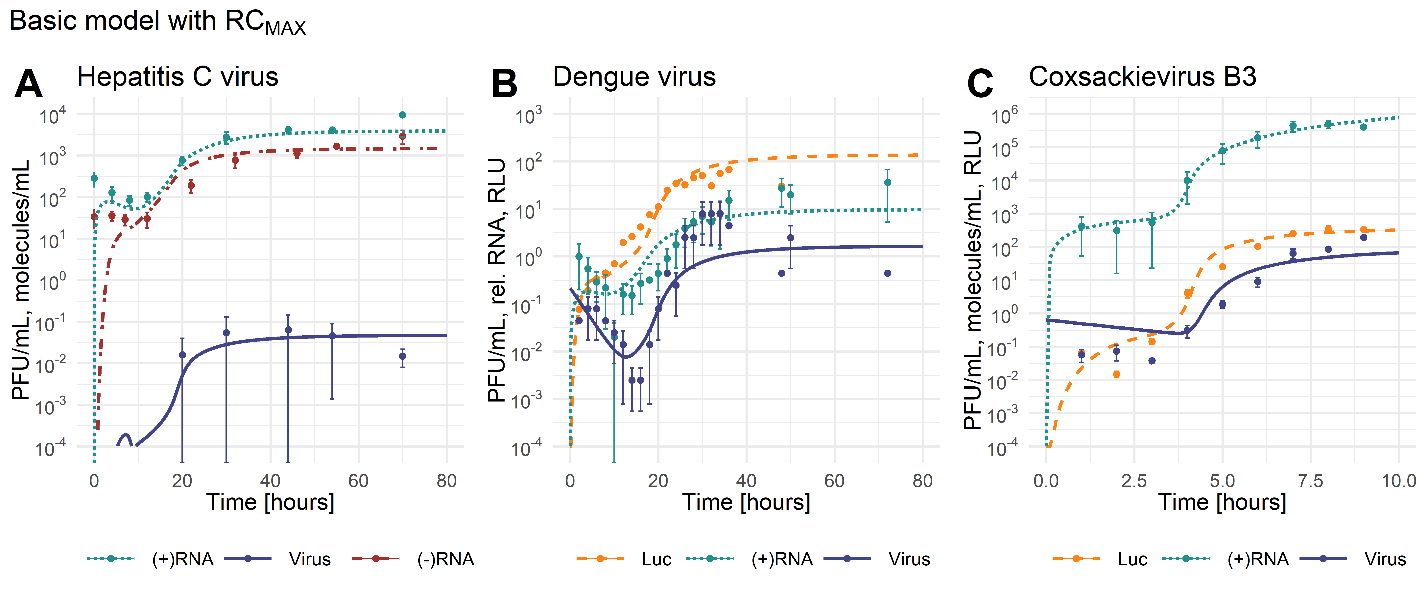


Fig B: Model fit of the basic model with $RC_{MAX}$. [green: (+)RNA = $R_{P}^{tot}= {(V}_{E}+ R_{V}+TC+ R_{RC}+ R_{DS}+ R_{IDS}+R_{R} {+ R}_{P})$, red: (-)RNA = $R_{M}^{tot}=(R_{DS}+R_{IDS})$, blue: A) Virus = $V^{tot}=V_{I}$, B) and C) Virus = $V^{tot}=(V+V_{I})$, yellow: Luc = L]


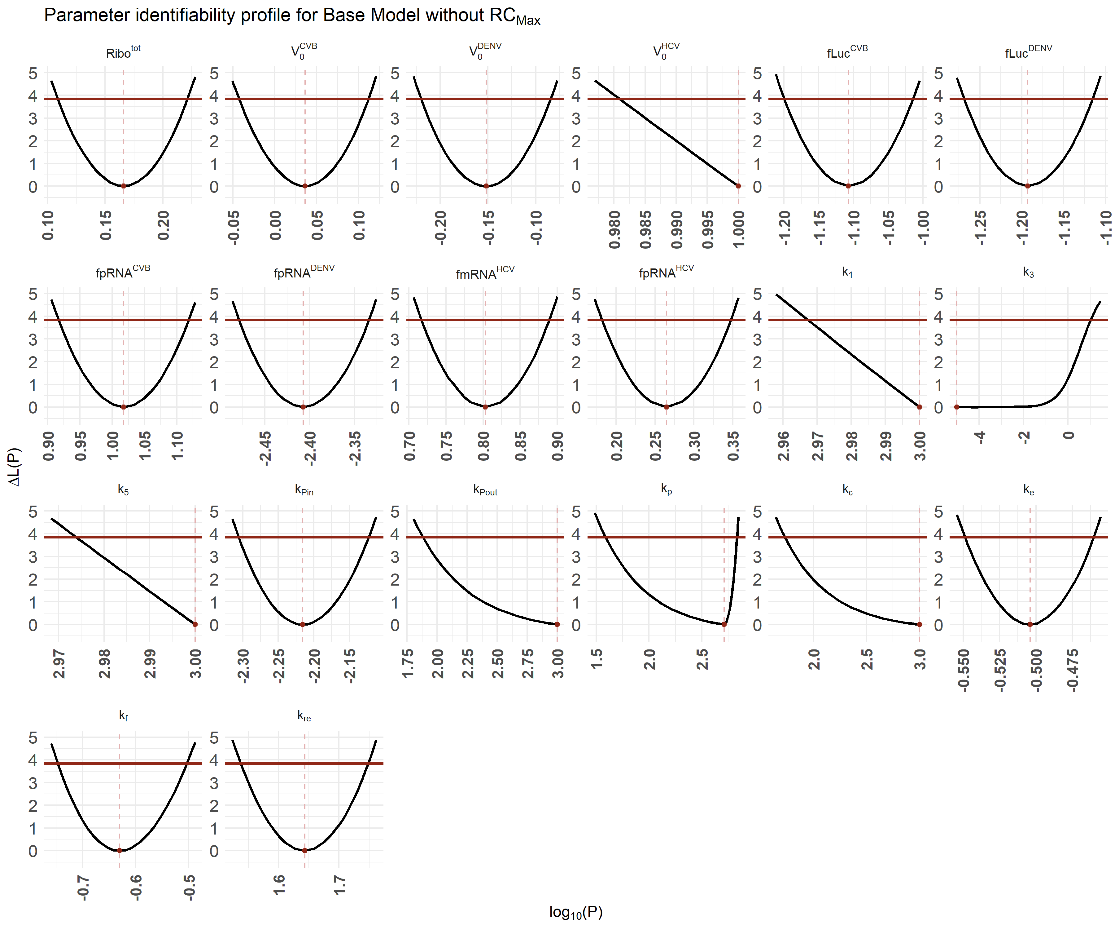


Fig C: Parameter profiles of estimated parameters of the model without a maximal number of replicase complexes.


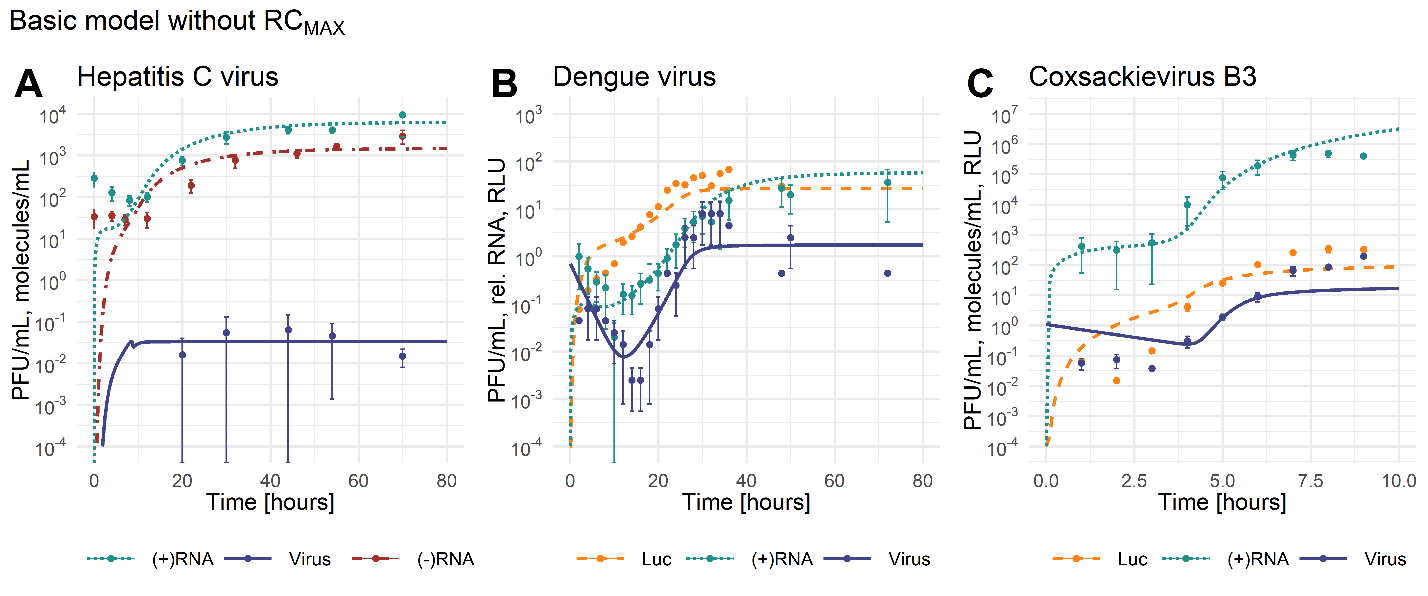


Fig D: Model fit for the basic model with $RC_{MAX}$. [green: (+)RNA = $R_{P}^{tot}= {(V}_{E}+ R_{V}+TC+ R_{RC}+ R_{DS}+ R_{IDS}+R_{R} {+ R}_{P})$ , red: (-)RNA = $R_{M}^{tot}=(R_{DS}+R_{IDS})$, blue: A) Virus = $V^{tot}=V_{I}$, B) and C) Virus = $V^{tot}=(V+V_{I})$, yellow: Luc = L]


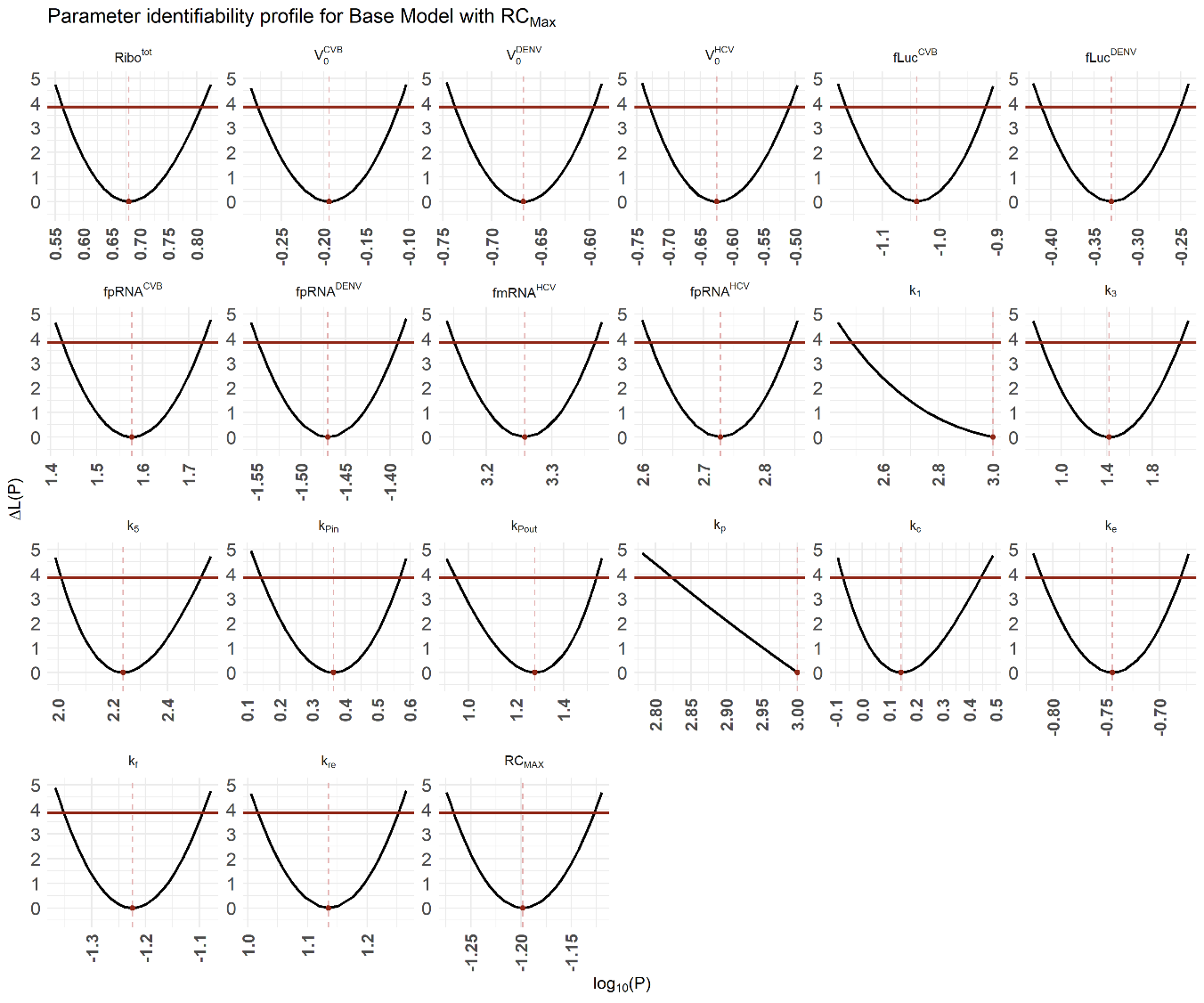


Fig E: Parameter profiles of estimated parameters of the model extended by the maximal number of replicase complexes.

## 2. Model selection of virus-specific differences

### 2.1 First round

In the first round of selecting virus-specific processes into our model, we tested all 13 different processes for model fit improvement and, thus, compared 13 different models. The best model fit and, thus, lowest AIC has been found by integrating a virus-specific ribosome concentration (Table B). Even though the model fit to the DENV data set visually worsened, the model fits to the HCV and CVB3 data sets improved, especially the very first hours post infection (Fig F). The virus specific parameters for the ribosomes were fully identifiable (Fig G) and, thus, integrated into the model for the following rounds of model selection.

Table B: Negative log likelihood (-LL), AIC and number of parameters (#p) for the first round of model selection. The best fit model is highlighted in green.

|  | Process |  | -LL | AIC | #p |
| --- | --- | --- | --- | --- | --- |
|  | Basic model with $RC_{MAX}$ |  | 1890 | 1982 | 46 |
| (i) | Virus entry and internalization | $k_{e}$ | 1680.3 | 1776.3 | 48 |
| (ii) | Release of the viral genome | $k_{f}$ | 1716.2 | 1812.2 | 48 |
| (iii) | Degradation of internalized virus within endosomes | $\mu_{VE}$ | 1833.5 | 1929.5 | 48 |
| (iv) | Formation of the translation initiation complex | $k_{1}$ | 1862.6 | 1958.6 | 48 |
| **(v)** | Total number of ribosomes available for viral RNA translation | $\boldsymbol{Rib}\boldsymbol{o}_{\boldsymbol{tot}}$ | **1611.6** | **1707.6** | **48** |
| (vi) | Polyprotein cleavage | $k_{c}$ | 1877.0 | 1973.0 | 48 |
| (vii) | Formation of replicase complex | $k_{Pin}$ | 1745.6 | 1841.6 | 48 |
| (viii) | Maximum number of replicase complexes | $RC_{MAX}$ | 1842.3 | 1938.3 | 48 |
| (ix) | Formation of replication intermediate complex | $k_{5}$ | 1834.9 | 1930.9 | 48 |
| (x) | Degradation of species within the replication organelle (RO) | $\mu_{RO}$ | 1868.7 | 1962.7 | 47 |
| (xi) | Further replication within the RO | $k_{3}$ | 1841.2 | 1937.2 | 48 |
| (xii) | Export of newly produced viral genomes from the RO to the site of translation | $k_{Pout}$ | 1857.1 | 1953.1 | 48 |
| (xiii) | Virus assembly and release | $k_{p}$ | 1809.8 | 1905.8 | 48 |


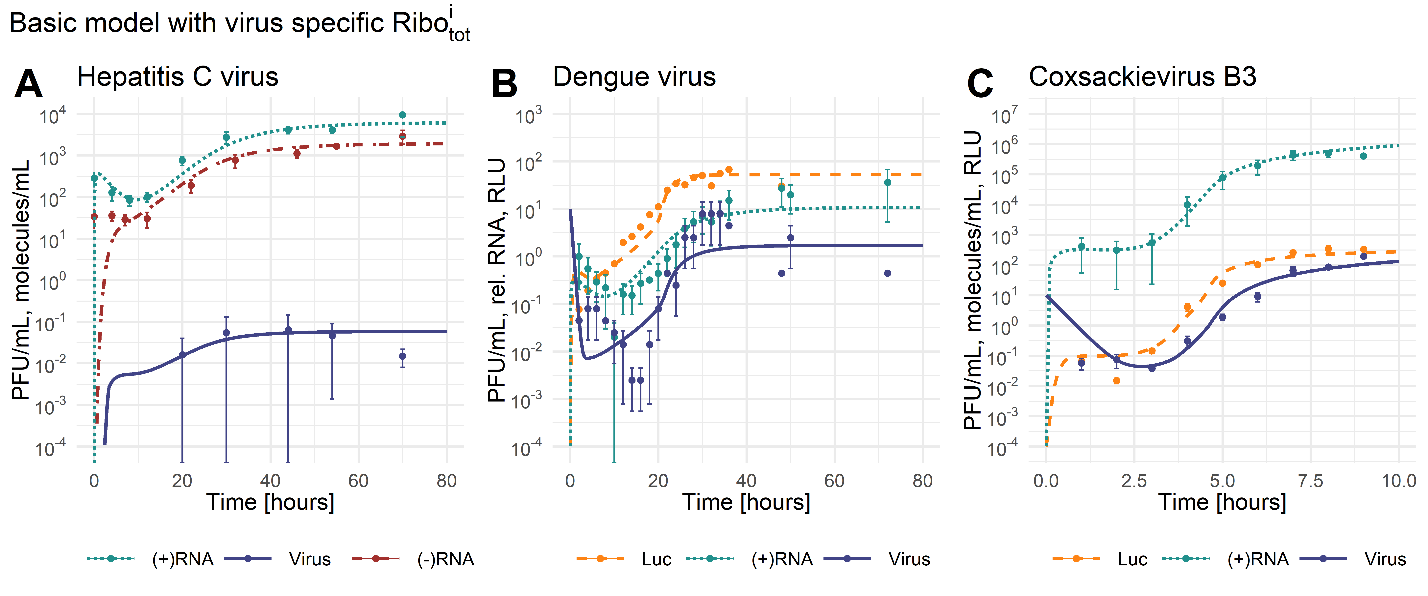


Fig F: Best fit of the model integrating the total number of ribosomes as virus specific. [green: (+)RNA = $R_{P}^{tot}= {(V}_{E}+ R_{V}+TC+ R_{RC}+ R_{DS}+ R_{IDS}+R_{R} {+ R}_{P})$ , red: (-)RNA = $R_{M}^{tot}=(R_{DS}+R_{IDS})$, blue: A) Virus = $V^{tot}=V_{I}$, B) and C) Virus = $V^{tot}=(V+V_{I})$, yellow: Luc = L]


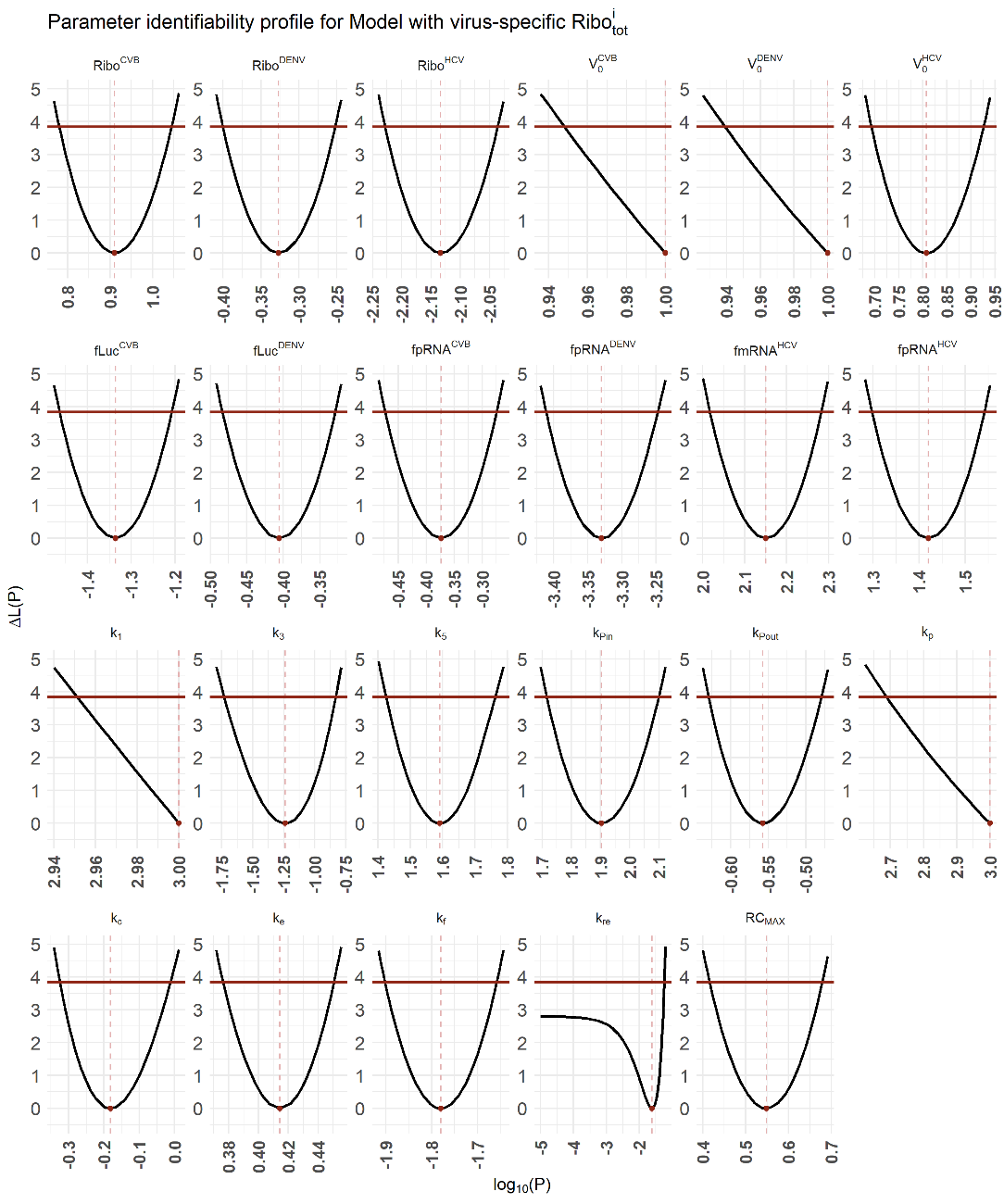


Fig G: Profiles of estimated parameters of the best fit model integrating a virus-specific availability of ribosomes.

### 2.2 Second Round

In the second round of model selection, we tested the remaining 12 processes for virus specificity. In the current round the viral uncoating process and, thus, fusion of the viral nucleocapsid with the endosomal membrane led to another tremendous improvement of the model fit (Fig H) and the AIC (Table C). While two of the virus-specific fusion parameters were identifiable (DENV and CVB3), that for HCV hit the upper estimation boundary (Fig I). Nevertheless, due to the tremendous improvement of the AIC, we added the fusion process as virus-specific into our final model.

Table C: Negative log likelihood (-LL), AIC and number of parameters (#p) for the second round of model selection. The best fit model of the current round is highlighted in green. The best fit model of the previous round is highlighted in yellow. Note that the yellow process is part of the model.

|  | Process |  | -LL | AIC | #p |
| --- | --- | --- | --- | --- | --- |
|  | Basic model with $RC_{MAX}$ |  | 1890 | 1982 | 46 |
| (i) | Virus entry and internalization | $k_{e}$ | 1325.7 | 1425.7 | 50 |
| **(ii)** | Release of the viral genome | $\boldsymbol{k}_{\boldsymbol{f}}$ | **1317.6** | **1417.6** | **50** |
| (iii) | Degradation of internalized virus within endosomes | $\mu_{VE}$ | 1500.2 | 1600.2 | 50 |
| (iv) | Formation of the translation initiation complex | $k_{1}$ | 1584.8 | 1684.8 | 50 |
| (v) | Total number of ribosomes available for viral RNA translation | $Ribo_{tot}$ | 1611.6 | 1707.6 | 48 |
| (vi) | Polyprotein cleavage | $k_{c}$ | 1555.1 | 1655.1 | 50 |
| (vii) | Formation of replicase complex | $k_{Pin}$ | 1452.6 | 1552.6 | 50 |
| (viii) | Maximum number of replicase complexes | $RC_{MAX}$ | 1497.5 | 1597.5 | 50 |
| (ix) | Formation of replication intermediate complex | $k_{5}$ | 1567.8 | 1667.8 | 50 |
| (x) | Degradation of species within the replication organelle (RO) | $\mu_{RO}$ | 1554.7 | 1652.7 | 49 |
| (xi) | Further replication within the RO | $k_{3}$ | 1548.1 | 1648.1 | 50 |
| (xii) | Export of newly produced viral genomes from the RO to the site of translation | $k_{Pout}$ | 1476.7 | 1576.7 | 50 |
| (xiii) | Virus assembly and release | $k_{p}$ | 1508.1 | 1608.1 | 50 |


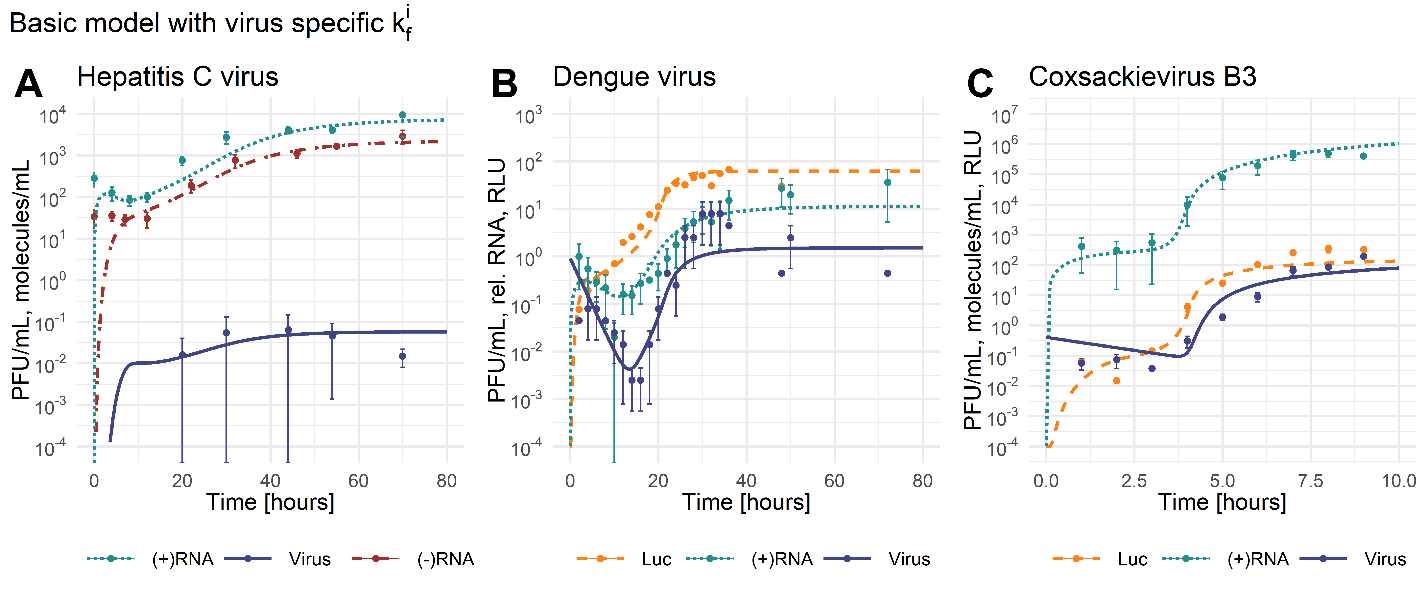


Fig H: Best fit of the model integrating the viral fusion process as virus specific. [green: (+)RNA = $R_{P}^{tot}= {(V}_{E}+ R_{V}+TC+ R_{RC}+ R_{DS}+ R_{IDS}+R_{R} {+ R}_{P})$ , red: (-)RNA = $R_{M}^{tot}=(R_{DS}+R_{IDS})$, blue: A) Virus = $V^{tot}=V_{I}$, B) and C) Virus = $V^{tot}=(V+V_{I})$, yellow: Luc = L]


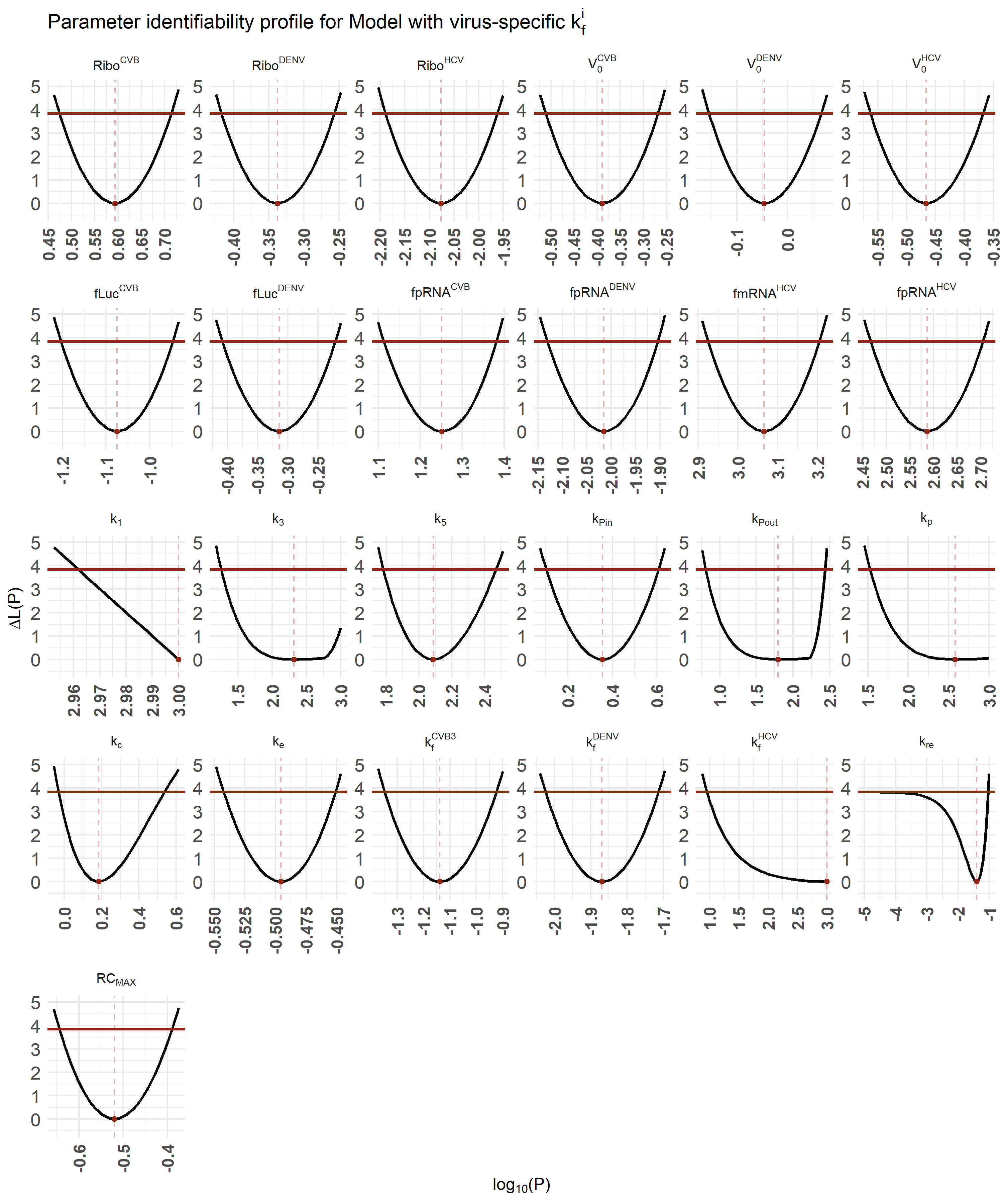


Fig I: Profiles of estimated parameters of the best fit model integrating the total number of ribosomes as virus specific.

### 2.3 Third Round

In the third round, from the remaining 11 processes viral RNA export out of the RO to the site of translation has been selected as virus-specific (Table D), which led to another improvement of the model fit (Fig J) and AIC (Table D). The virus specific parameter values for $k_{Pout}^{i}$ were all identifiable thus integrated into the model (Fig K).

Table D: Negative log likelihood (-LL), AIC and number of parameters (#p) for the third round of model selection. The best fit model of the current round is highlighted in green. The best fit models of the previous rounds are highlighted in yellow. Note that the yellow processes are part of the model.

|  | Process |  | -LL | AIC | #p |
| --- | --- | --- | --- | --- | --- |
|  | Basic model with $RC_{MAX}$ |  | 1890 | 1982 | 46 |
| (i) | Virus entry and internalization | $k_{e}$ | 1191.7 | 1295.7 | 52 |
| (ii) | Release of the viral genome | $k_{f}$ | 1317.6 | 1417.6 | 50 |
| (iii) | Degradation of internalized virus within endosomes | $\mu_{VE}$ | 1310.7 | 1414.7 | 52 |
| (iv) | Formation of the translation initiation complex | $k_{1}$ | 1174.2 | 1278.2 | 52 |
| (v) | Total number of ribosomes available for viral RNA translation | $Ribo_{tot}$ | 1611.6 | 1707.6 | 48 |
| (vi) | Polyprotein cleavage | $k_{c}$ | 1208.1 | 1312.1 | 52 |
| (vii) | Formation of replicase complex | $k_{Pin}$ | 1282.4 | 1386.4 | 52 |
| (viii) | Maximum number of replicase complexes | $RC_{MAX}$ | 1112.5 | 1216.5 | 52 |
| (ix) | Formation of replication intermediate complex | $k_{5}$ | 1163.1 | 1267.1 | 52 |
| (x) | Degradation of species within the replication organelle (RO) | $\mu_{RO}$ | 1185.0 | 1287.0 | 51 |
| (xi) | Further replication within the RO | $k_{3}$ | 1158.4 | 1262.4 | 52 |
| **(xii)** | Export of newly produced viral genomes from the RO to the site of translation | $\boldsymbol{k}_{\boldsymbol{Pout}}$ | **1109.2** | **1213.2** | **52** |
| (xiii) | Virus assembly and release | $k_{p}$ | 1287.4 | 1391.4 | 52 |


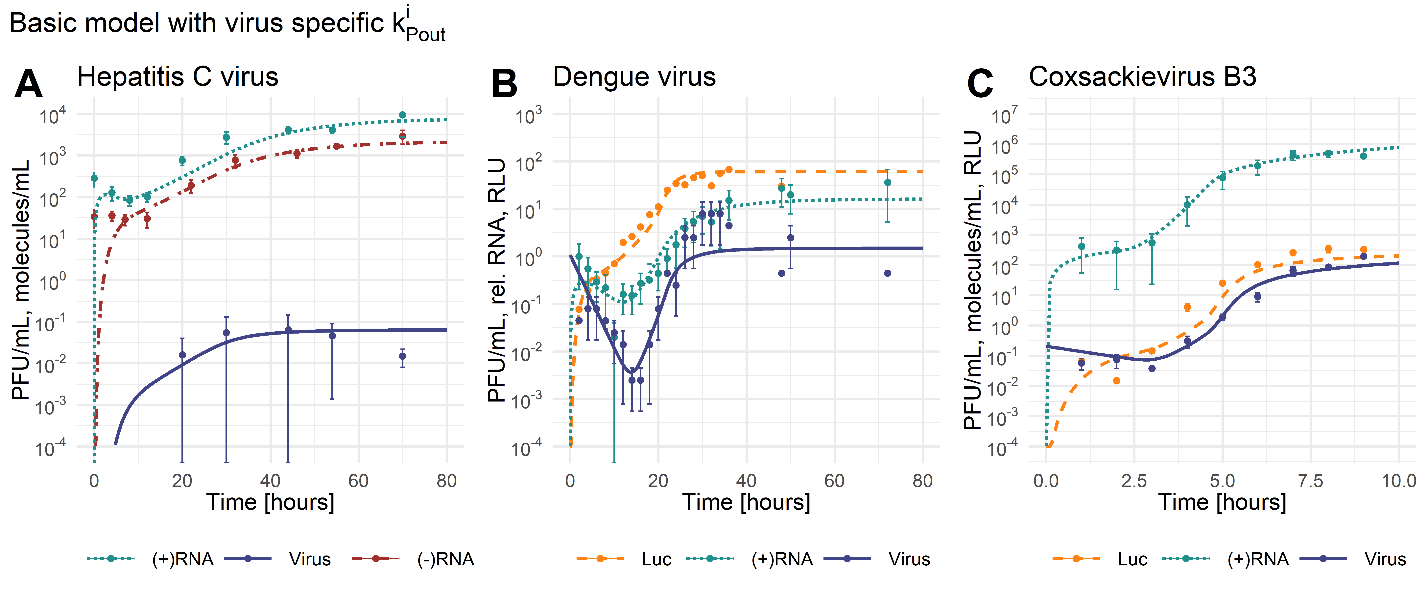


Fig J: Best fit of the model integrating the viral RNA export from the RO to the site of translation as virus specific. [green: (+)RNA = $R_{P}^{tot}= {(V}_{E}+ R_{V}+TC+ R_{RC}+ R_{DS}+ R_{IDS}+R_{R} {+ R}_{P})$ , red: (-)RNA = $R_{M}^{tot}=(R_{DS}+R_{IDS})$, blue: A) Virus = $V^{tot}=V_{I}$, B) and C) Virus = $V^{tot}=(V+V_{I})$, yellow: Luc = L]


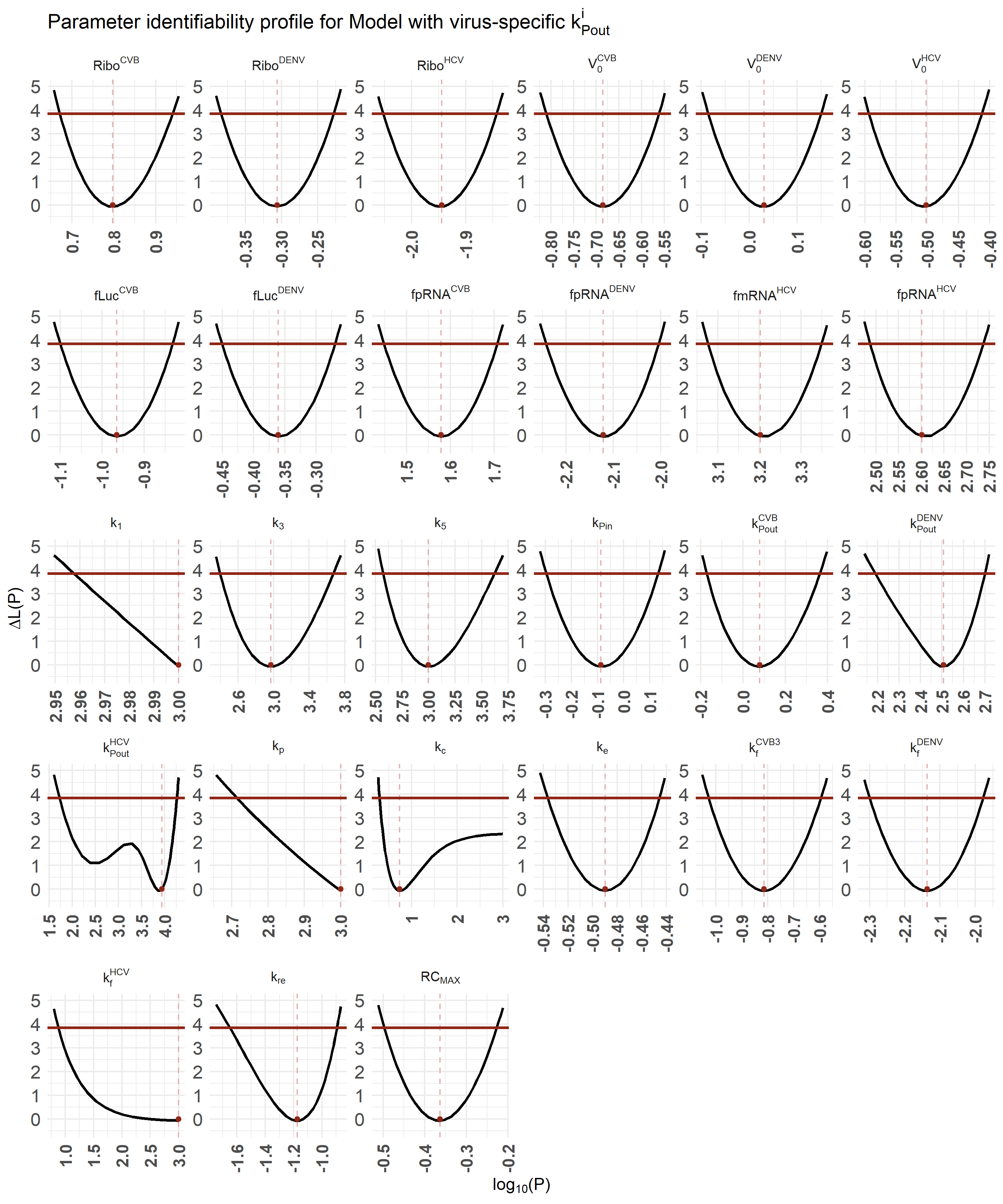


Fig K: Profiles of estimated parameters of the best fit model integrating the viral RNA export from the RO to the site of translation as virus specific.

### 2.4 Fourth Round

In the fourth round, virus specific entry showed the lowest AIC from the remaining 10 processes (Fig L and Table E). Similar to viral fusion, the parameter values were identifiable for DENV and CVB3, however, the parameter profile hit the upper estimation boundary (Fig M).

Table E: Negative log likelihood (-LL), AIC and number of parameters (#p) for the fourth round of model selection. The best fit model of the current round is highlighted in green. The best fit models of the previous rounds are highlighted in yellow. Note that the yellow processes are part of the model.

|  | Process |  | -LL | AIC | #p |
| --- | --- | --- | --- | --- | --- |
|  | Basic model with $RC_{MAX}$ |  | 1890 | 1982 | 46 |
| **(i)** | Virus entry and internalization | $\boldsymbol{k}_{\boldsymbol{e}}$ | **1045.7** | **1153.7** | **54** |
| (ii) | Release of the viral genome | $k_{f}$ | 1317.6 | 1417.6 | 50 |
| (iii) | Degradation of internalized virus within endosomes | $\mu_{VE}$ | 1109.0 | 1217.0 | 54 |
| (iv) | Formation of the translation initiation complex | $k_{1}$ | 1103.7 | 1211.7 | 54 |
| (v) | Total number of ribosomes available for viral RNA translation | $Ribo_{tot}$ | 1611.6 | 1707.6 | 48 |
| (vi) | Polyprotein cleavage | $k_{c}$ | 1095.4 | 1203.4 | 54 |
| (vii) | Formation of replicase complex | $k_{Pin}$ | 1074.1 | 1182.1 | 54 |
| (viii) | Maximum number of replicase complexes | $RC_{MAX}$ | 1087.2 | 1195.2 | 54 |
| (ix) | Formation of replication intermediate complex | $k_{5}$ | 1100.0 | 1208.0 | 54 |
| (x) | Degradation of species within the replication organelle (RO) | $\mu_{RO}$ | 1061.2 | 1167.2 | 53 |
| (xi) | Further replication within the RO | $k_{3}$ | 1092.5 | 1200.5 | 54 |
| (xii) | Export of newly produced viral genomes from the RO to the site of translation | $k_{Pout}$ | 1109.2 | 1213.2 | 52 |
| (xiii) | Virus assembly and release | $k_{p}$ | 1107.2 | 1215.2 | 54 |


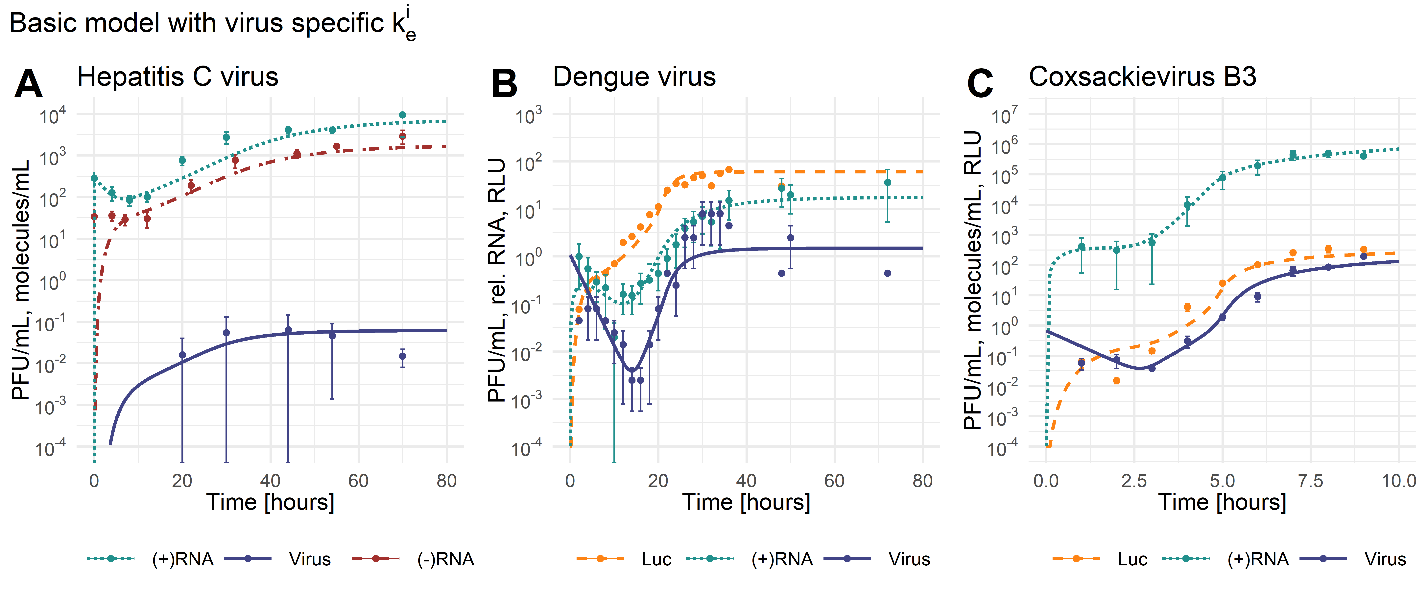
Fig L: Best fit of the model integrating the viral entry process as virus specific. [green: (+)RNA = $R_{P}^{tot}= {(V}_{E}+ R_{V}+TC+ R_{RC}+ R_{DS}+ R_{IDS}+R_{R} {+ R}_{P})$ , red: (-)RNA = $R_{M}^{tot}=(R_{DS}+R_{IDS})$, blue: A) Virus = $V^{tot}=V_{I}$, B) and C) Virus = $V^{tot}=(V+V_{I})$, yellow: Luc = L]


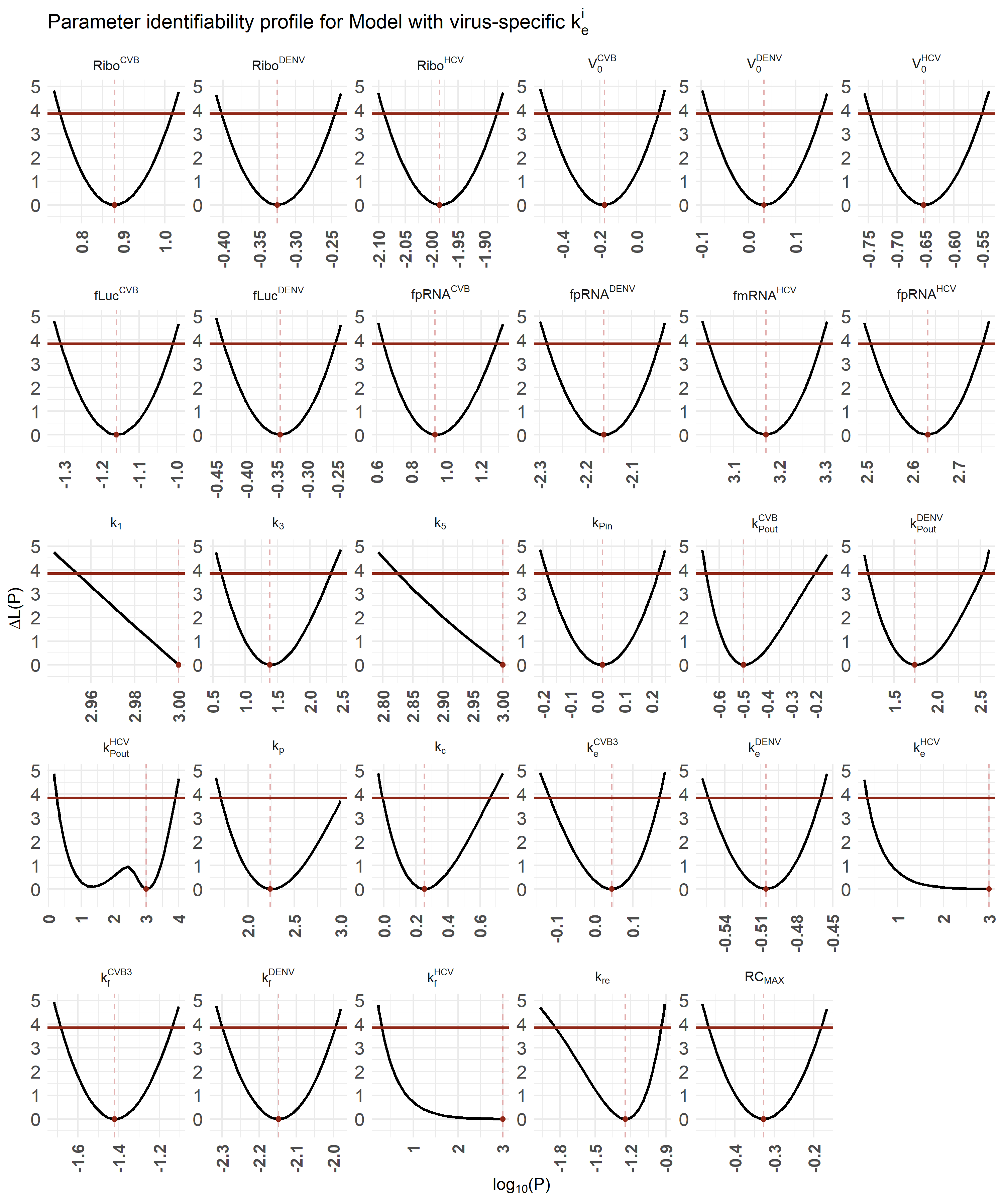


Fig M: Profiles of estimated parameters of the best fit model integrating the viral entry process as virus specific

### 2.5 Fifth Round

In the fifth round of model selection, two processes showed comparable AIC values with ΔAIC = 0.8 (Table F, Figs N and P). Therefore, we tested the identifiability of both processes: polyprotein cleavage and the formation of replicase complexes. While the virus-specific polyprotein cleavage was only identifiable for CVB3, the parameter profiles hit the upper estimation boundary for HCV and DENV (Fig O). However, the virus-specific formation of the replicase complexes was identifiable for all three viruses and, thus, we added that process into our final model (Fig Q).

Table F: Negative log likelihood (-LL), AIC and number of parameters (#p) for the fifth round of model selection. The best fit models of the current round are highlighted in green. The best fit models of the previous rounds are highlighted in yellow. Note that the yellow processes are part of the model.

|  | Process |  | -LL | AIC | #p |
| --- | --- | --- | --- | --- | --- |
|  | Basic model with $RC_{MAX}$ |  | 1890 | 1982 | 46 |
| (i) | Virus entry and internalization | $k_{e}$ | 1045.7 | 1153.7 | 54 |
| (ii) | Release of the viral genome | $k_{f}$ | 1317.6 | 1417.6 | 50 |
| (iii) | Degradation of internalized virus within endosomes | $\mu_{VE}$ | 1039.3 | 1151.3 | 56 |
| (iv) | Formation of the translation initiation complex | $k_{1}$ | 1029.5 | 1141.5 | 56 |
| (v) | Total number of ribosomes available for viral RNA translation | $Ribo_{tot}$ | 1611.6 | 1707.6 | 48 |
| **(vi)** | Polyprotein cleavage | $\boldsymbol{k}_{\boldsymbol{c}}$ | **1013.6** | **1125.6** | **56** |
| **(vii)** | Formation of replicase complex | $\boldsymbol{k}_{\boldsymbol{Pin}}$ | **1014.4** | **1126.4** | **56** |
| (viii) | Maximum number of replicase complexes | $RC_{MAX}$ | 1044.4 | 1156.4 | 56 |
| (ix) | Formation of replication intermediate complex | $k_{5}$ | 1045.7 | 1157.7 | 56 |
| (x) | Degradation of species within the replication organelle (RO) | $\mu_{RO}$ | 1017.9 | 1127.9 | 55 |
| (xi) | Further replication within the RO | $k_{3}$ | 1041.7 | 1153.7 | 56 |
| (xii) | Export of newly produced viral genomes from the RO to the site of translation | $k_{Pout}$ | 1109.2 | 1213.2 | 52 |
| (xiii) | Virus assembly and release | $k_{p}$ | 1041.6 | 1153.6 | 56 |


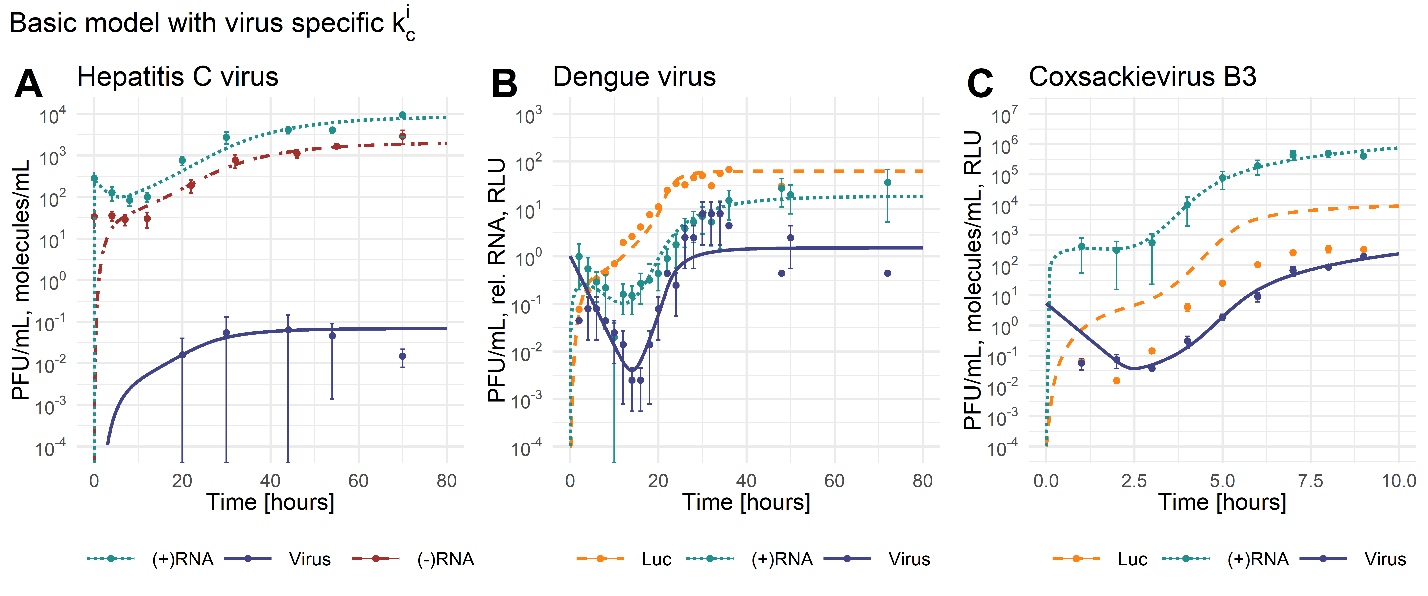


Fig N: Best fit of the model integrating polyprotein cleavage as virus specific. [green: (+)RNA = $R_{P}^{tot}= {(V}_{E}+ R_{V}+TC+ R_{RC}+ R_{DS}+ R_{IDS}+R_{R} {+ R}_{P})$ , red: (-)RNA = $R_{M}^{tot}=(R_{DS}+R_{IDS})$, blue: A) Virus = $V^{tot}=V_{I}$, B) and C) Virus = $V^{tot}=(V+V_{I})$, yellow: Luc = L]


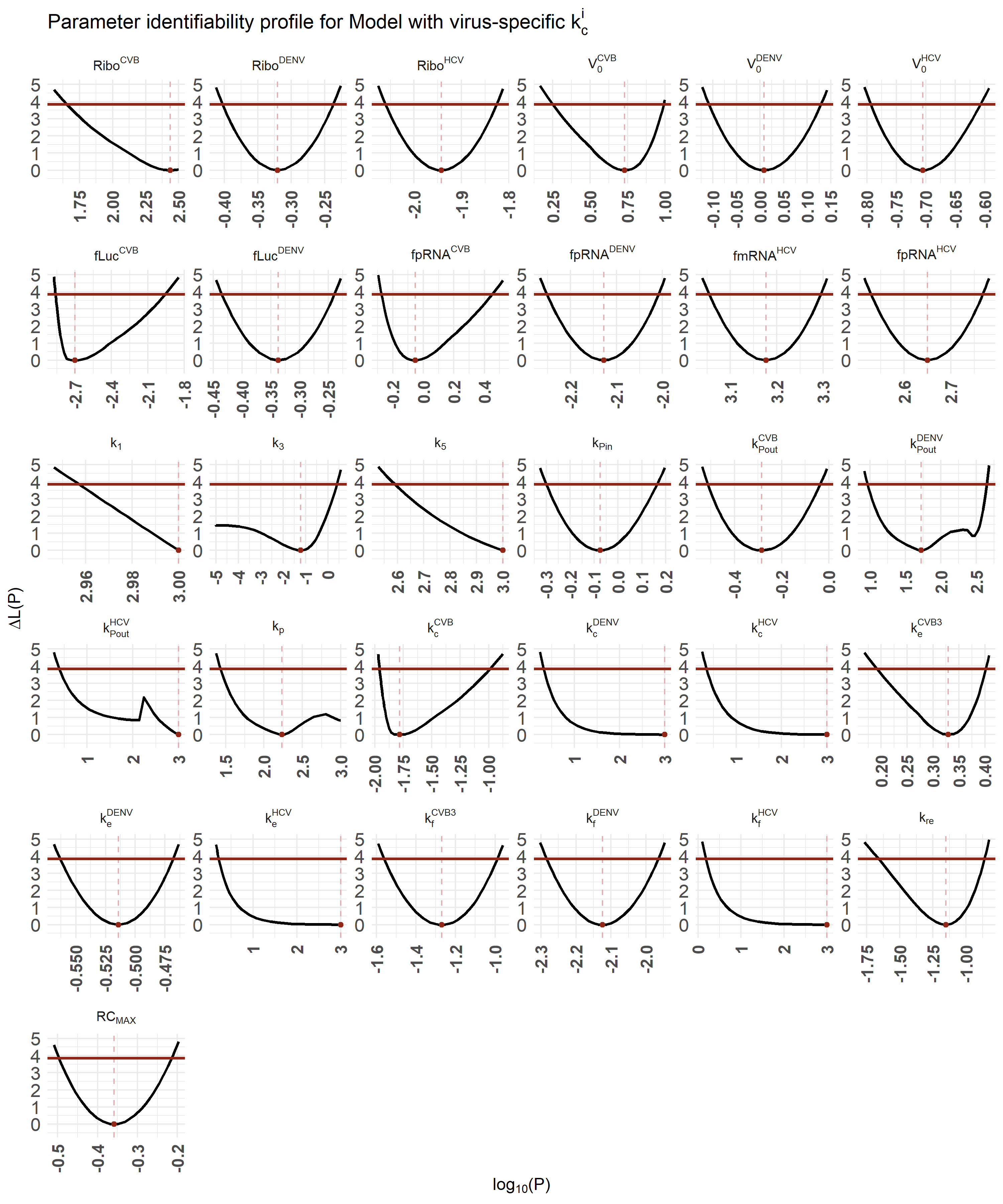


Fig O: Profiles of estimated parameters of the best fit model integrating polyprotein cleavage as virus specific.


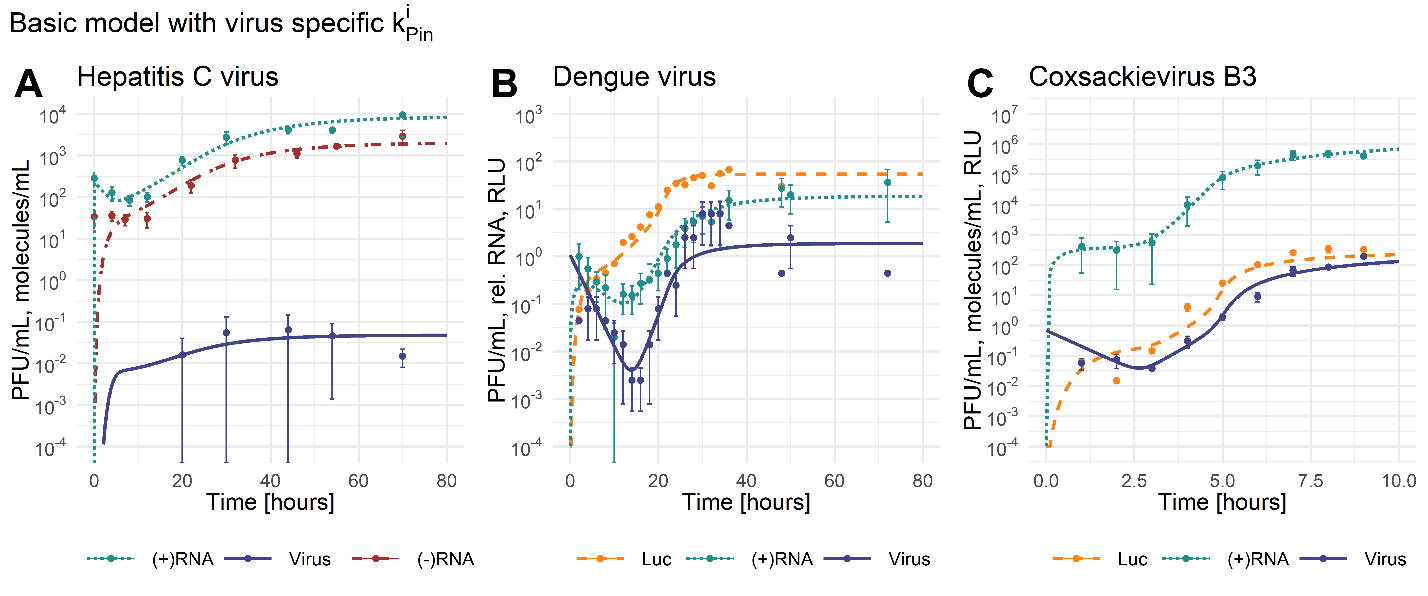


Fig P: Best fit of the model integrating the formation of the replicase complex as virus specific. [green: (+)RNA = $R_{P}^{tot}= {(V}_{E}+ R_{V}+TC+ R_{RC}+ R_{DS}+ R_{IDS}+R_{R} {+ R}_{P})$ , red: (-)RNA = $R_{M}^{tot}=(R_{DS}+R_{IDS})$, blue: A) Virus = $V^{tot}=V_{I}$, B) and C) Virus = $V^{tot}=(V+V_{I})$, yellow: Luc = L]


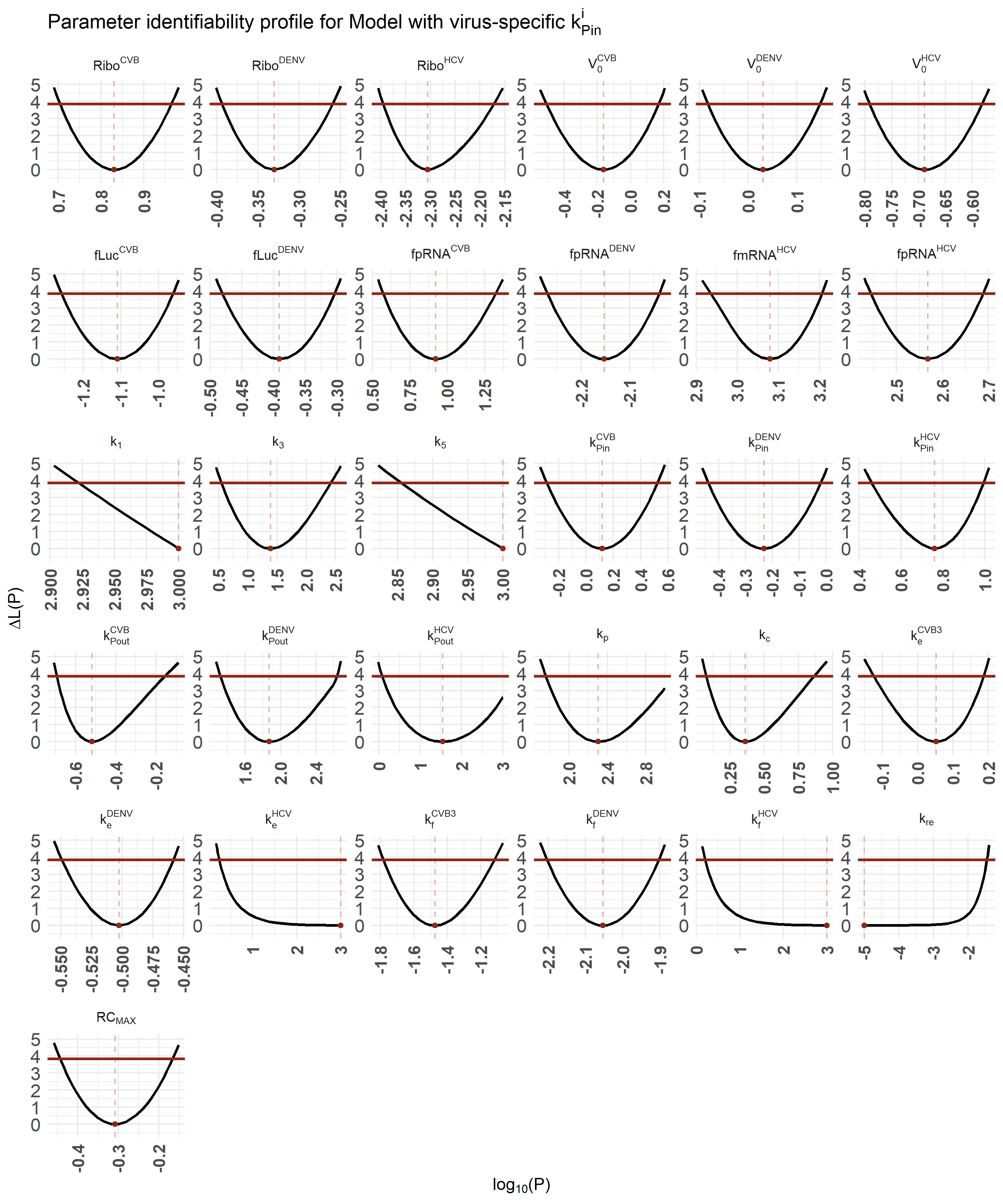


Fig Q: Profiles of estimated parameters of the best fit model integrating the formation of the replicase complex as virus specific.

### 2.6 Sixth Round

In the sixth round, again two comparable model fits have been observed with ΔAIC = 0.9 (Table G, Figs R and T). As in the previous round, we tested the parameter identifiability for both processes: virus specific polyprotein cleavage rate and degradation of species within the replication compartment. Similar to the previous round, the virus-specific parameters for polyprotein cleavage were identifiable for CVB3 while their parameter profiles hit the upper estimation boundary for HCV and DENV (Fig U). However, the virus-specific parameter values for the degradation of species within the RO were not identifiable for CVB3 and hit the lower estimation boundary for DENV (Fig S). Note that here the virus-specific degradation rate was fixed for HCV to a literature value (see Table 2 Main text). Hence, we stopped after five rounds model selection and did not integrate more virus-specific processes into our model due to increasing uncertainty.

Table G: Negative log likelihood (-LL), AIC and number of parameters (#p) for the sixth round of model selection. The best fit model of the current round is highlighted in green. The best fit models of the previous rounds are highlighted in yellow. Note that the yellow processes are part of the model.

|  | Process |  | -LL | AIC | #p |
| --- | --- | --- | --- | --- | --- |
|  | Basic model with $RC_{MAX}$ |  | 1890 | 1982 | 46 |
| (i) | Virus entry and internalization | $k_{e}$ | 1045.7 | 1153.7 | 54 |
| (ii) | Release of the viral genome | $k_{f}$ | 1317.6 | 1417.6 | 50 |
| (iii) | Degradation of internalized virus within endosomes | $\mu_{VE}$ | 1008.2 | 1124.2 | 58 |
| (iv) | Formation of the translation initiation complex | $k_{1}$ | 990.9 | 1106.9 | 58 |
| (v) | Total number of ribosomes available for viral RNA translation | $Ribo_{tot}$ | 1611.6 | 1707.6 | 48 |
| **(vi)** | Polyprotein cleavage | $\boldsymbol{k}_{\boldsymbol{c}}$ | **971.7** | **1087.7** | **58** |
| (vii) | Formation of replicase complex | $k_{Pin}$ | 1014.4 | 1126.4 | 56 |
| (viii) | Maximum number of replicase complexes | $RC_{MAX}$ | 1007.3 | 1123.3 | 58 |
| (ix) | Formation of replication intermediate complex | $k_{5}$ | 1007.2 | 1123.2 | 58 |
| **(x)** | Degradation of species within the replication organelle (RO) | $\boldsymbol{\mu}_{\boldsymbol{RO}}$ | **972.8** | **1086.8** | **57** |
| (xi) | Further replication within the RO | $k_{3}$ | 1011.0 | 1127.0 | 58 |
| (xii) | Export of newly produced viral genomes from the RO to the site of translation | $k_{Pout}$ | 1109.2 | 1213.2 | 52 |
| (xiii) | Virus assembly and release | $k_{p}$ | 1010.5 | 1126.5 | 58 |


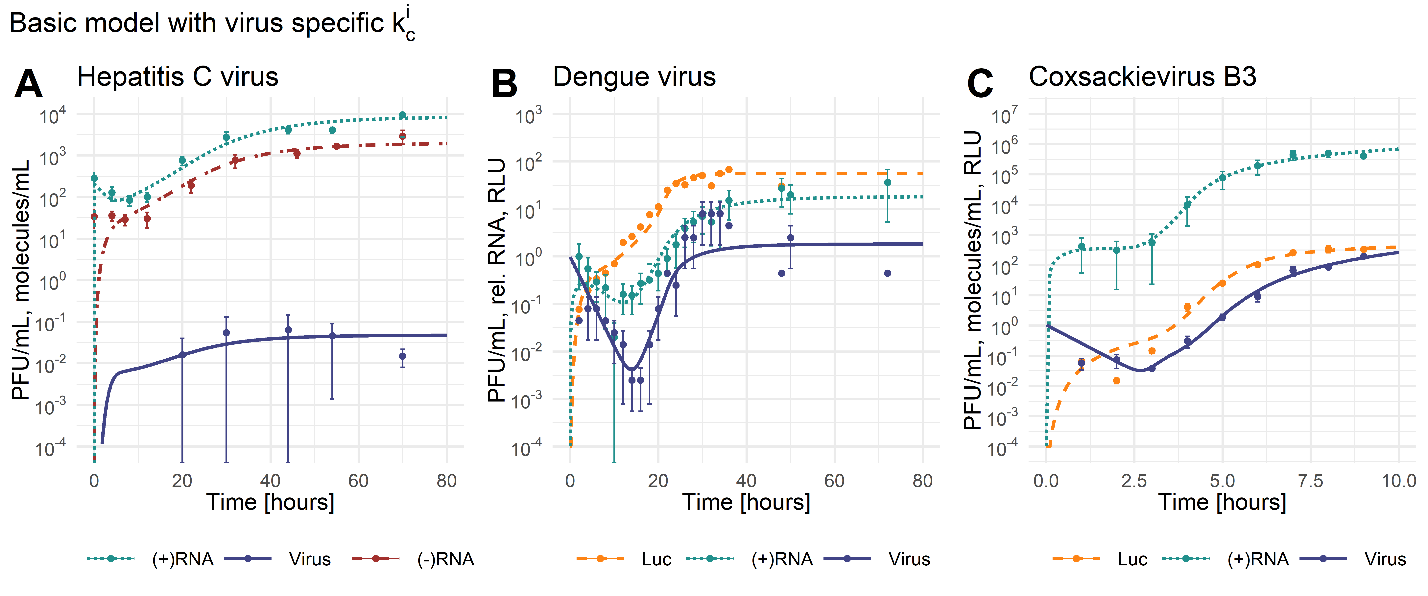


Fig R: Best fit of the model integrating the degradation rate of species within the replication complex as virus specific. [green: (+)RNA = $R_{P}^{tot}= {(V}_{E}+ R_{V}+TC+ R_{RC}+ R_{DS}+ R_{IDS}+R_{R} {+ R}_{P})$ , red: (-)RNA = $R_{M}^{tot}=(R_{DS}+R_{IDS})$, blue: A) Virus = $V^{tot}=V_{I}$, B) and C) Virus = $V^{tot}=(V+V_{I})$, yellow: Luc = L]


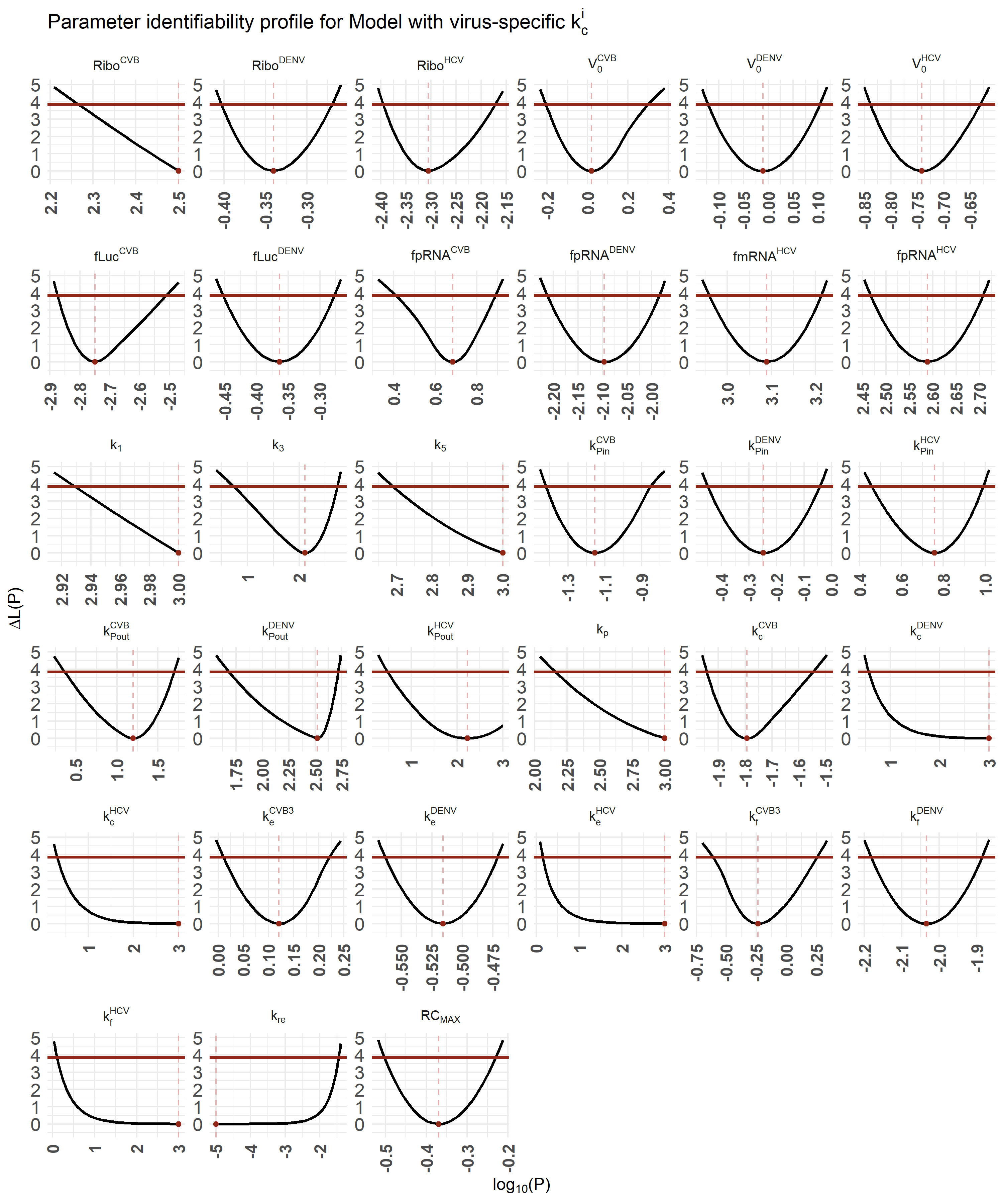


Fig S: Profiles of estimated parameters in the best fit model integrating the degradation rate of species within the replication complex as virus specific.


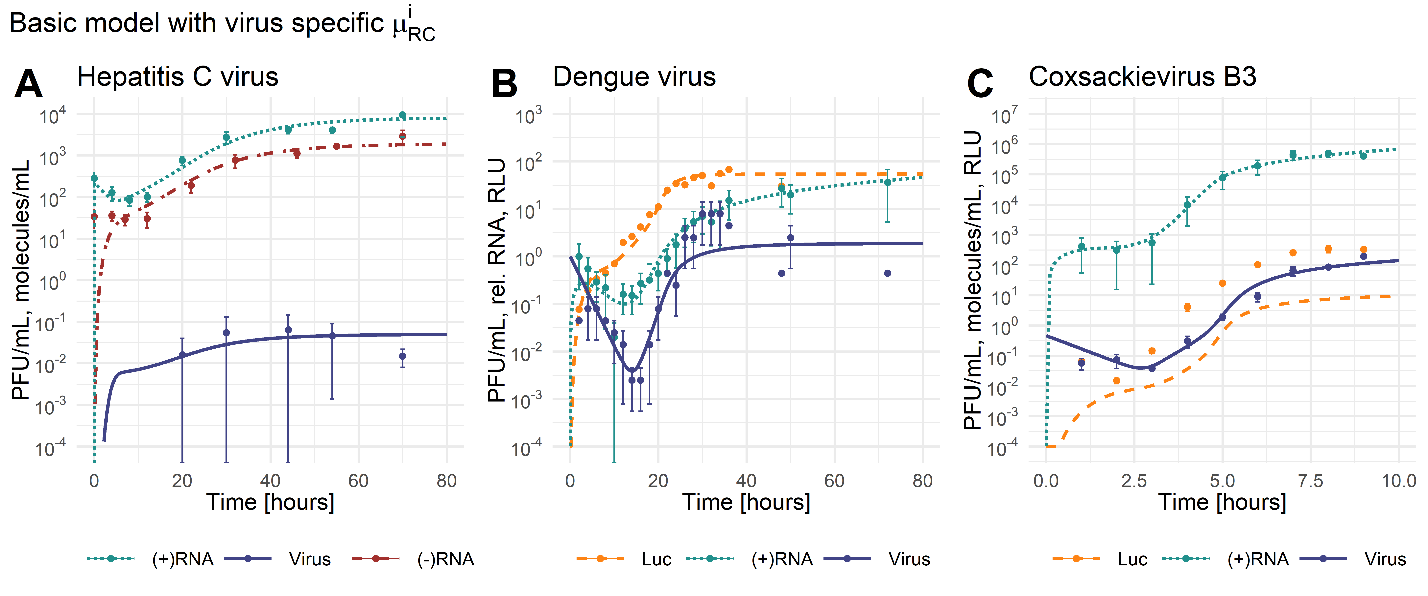


Fig T: Best fit of the model integrating the polyprotein cleavage as virus specific. [green: (+)RNA = $R_{P}^{tot}= {(V}_{E}+ R_{V}+TC+ R_{RC}+ R_{DS}+ R_{IDS}+R_{R} {+ R}_{P})$ , red: (-)RNA = $R_{M}^{tot}=(R_{DS}+R_{IDS})$, blue: A) Virus = $V^{tot}=V_{I}$, B) and C) Virus = $V^{tot}=(V+V_{I})$, yellow: Luc = L]


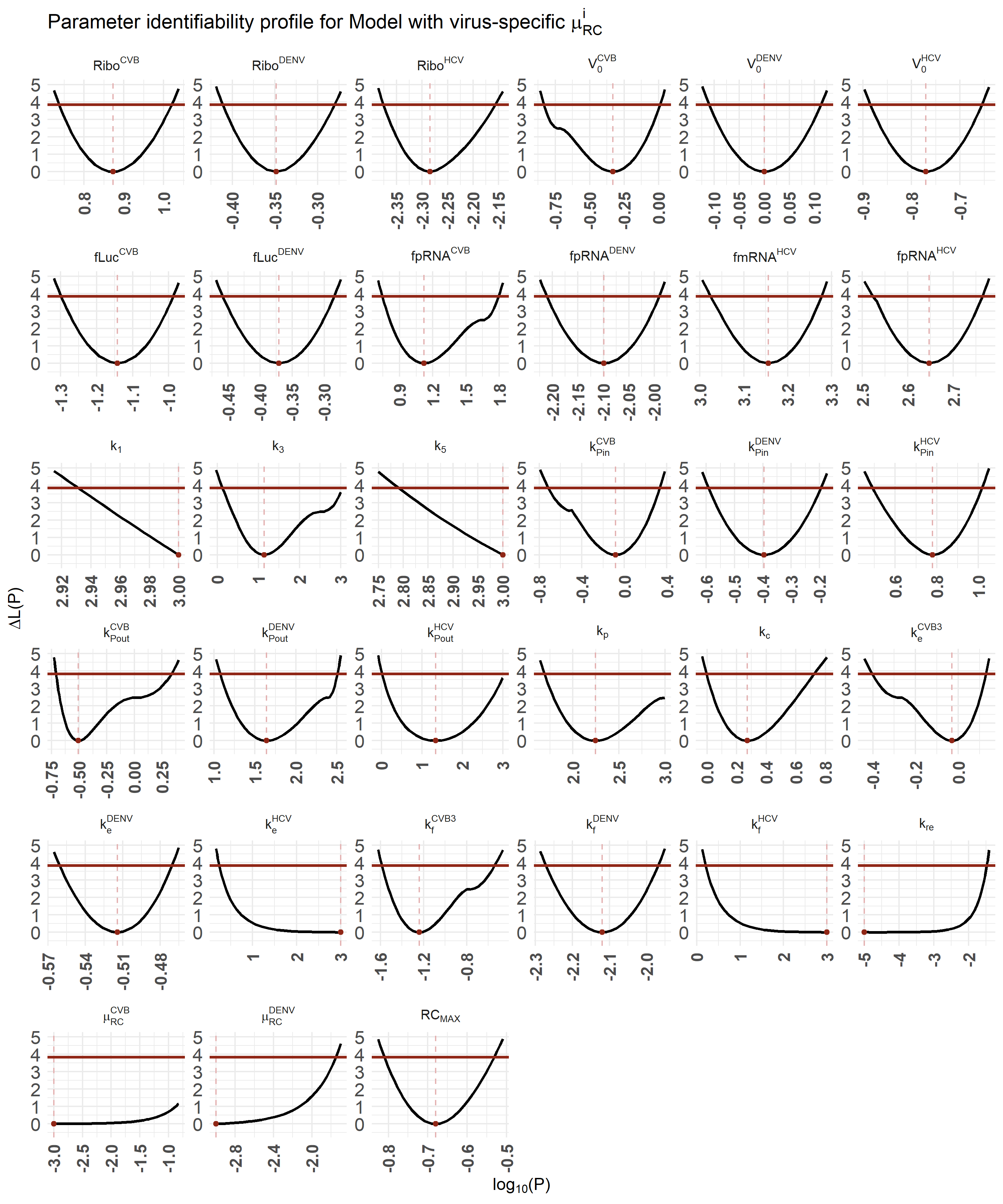


Fig U: Profiles of estimated parameters in the best fit model integrating polyprotein cleavage as virus specific.

### 2.7 Summary

Table H summarizes the single rounds of model selection and the stepwise improvement of the AIC. We integrated the top five processes as virus-specific into our model, which showed a high degree of identifiability. However, as shown in the table by integrating more virus-specific processes the AIC further improves but also the number of parameters that worsens the model identifiability.

Table H: Negative log likelihood (-LL), AIC and number of parameters (#p) for the sixth round of model selection. The best fit model of the current round is highlighted in green. The best fit models of the previous rounds are highlighted in yellow.

|  | **virus-specific process** |  | **-LL** | **AIC** | **#p** |
| --- | --- | --- | --- | --- | --- |
| **Round 1** | **total number of ribosomes available for translation** | $\boldsymbol{Rib}\boldsymbol{o}_{\boldsymbol{tot}}$ | **1611.6** | **1708** | **48** |
| **Round 2** | **fusion** | $\boldsymbol{k}_{\boldsymbol{f}}$ | **1317.6** | **1418** | **50** |
| **Round 3** | **export of newly produced viral genome of the replication organelle to the site of translation** | $\boldsymbol{k}_{\boldsymbol{Pout}}$ | **1109.2** | **1213** | **54** |
| **Round 4** | **virus entry** | $\boldsymbol{k}_{\boldsymbol{e}}$ | **1045.7** | **1154** | **54** |
| **Round 5** | **formation of replicase complex** | $\boldsymbol{k}_{\boldsymbol{Pin}}$ | **1014.4** | **1126** | **56** |
| **AIC TOP 5** |  |  |  | **1126** |  |
| Round 6 | degradation of species within the replication organelle | $\mu_{RO}$ | 972.8359 | 1086.836 | 57 |
| Round 7 | polyprotein cleavage | $k_{c}$ | 926.9 | 1044.9 | 59 |
| Round 8 | degradation of internalized virus within endosomes | $\mu_{VE}$ | 915.5 | 1037.5 | 61 |
| Round 9 | formation of replication intermediate complex | $k_{5}$ | 910.8 | 1036.8 | 63 |
| Round 10 | maximum number of replicase complexes | $RC_{MAX}$ | 898.6 | 1028.6 | 65 |
| AIC TOP 10 |  |  |  | 1029 |  |

1. Raue A, Steiert B, Schelker M, Kreutz C, Maiwald T, Hass H, et al. Data2Dynamics: a modeling environment tailored to parameter estimation in dynamical systems. Bioinformatics. 2015;31: 3558–3560. doi:10.1093/bioinformatics/btv405

2. Raue A, Schilling M, Bachmann J, Matteson A, Schelker M, Schelke M, et al. Lessons learned from quantitative dynamical modeling in systems biology. PLoS One. 2013;8: e74335. doi:10.1371/journal.pone.0074335

3. Burnham KP, Anderson DR. Model selection and multimodel inference: A practical information-theoretic approach. Springer. 2002; 488. doi:10.2307/3802723

4. Kreutz C, Raue A, Kaschek D, Timmer J. Profile likelihood in systems biology. FEBS Journal. 2013. pp. 2564–2571. doi:10.1111/febs.12276

5. Raue A, Kreutz C, Maiwald T, Bachmann J, Schilling M, Klingmüller U, et al. Structural and practical identifiability analysis of partially observed dynamical models by exploiting the profile likelihood. Bioinformatics. 2009;25: 1923–1929. doi:10.1093/bioinformatics/btp358

6. Binder M, Sulaimanov N, Clausznitzer D, Schulze M, Hüber CM, Lenz SM, et al. Replication vesicles are load- and choke-points in the hepatitis C virus lifecycle. PLoS Pathog. 2013;9: e1003561. doi:10.1371/journal.ppat.1003561

7. Zitzmann C, Schmid B, Ruggieri A, Perelson AS, Binder M, Bartenschlager R, et al. A coupled mathematical model of the intracellular replication of dengue virus and the host cell immune response to infection. Front Microbiol. 2020;11: 725. doi:10.3389/fmicb.2020.00725
