# Supplementary material for: Mathematical modeling of plus-strand RNA virus replication to identify broad-spectrum antiviral treatment strategies
Source: PLoS Comput Biol. 2023 Apr 4;19(4):e1010423. doi: 10.1371/journal.pcbi.1010423 (PMC10104377; doi:10.1371/journal.pcbi.1010423)
Supplement: S3 Text — (DOCX) [file pcbi.1010423.s003.docx]

# S3 Supporting text: Comparison of the plus-strand RNA virus replication model with our previous models

The current study is based on our previous publications modeling the life cycles of HCV [1] and DENV [2]. Both models describe vRNA translation and synthesis as core processes. Even though the core models are similar, there are crucial differences between the models.

First, our previously published HCV model was developed for a transfection experiment of sub-genomic RNA that does not contain genes for structural proteins and, thus, cannot produce infectious virus. However, for our recently published DENV model, we extended the model by cell infection—virus entry and vRNA genome release—and virus assembly and release.

Second, our previous HCV and DENV models were developed to study the viral life cycles in different cell lines. Our previous HCV model addressed differences in the HCV life cycle in high- and low-permissive cell lines by integrating host factors involved in the RC formation. However, our previous DENV model addressed the differences in the DENV life cycle in the absence and presence of the host cellular immune response—in two different cell lines. This has been achieved by integrating host factors that account for cell-line differences, such as the RC formation and virus assembly or adapting model parameters in the DENV life cycle, that are affected by the host cellular immune response, e.g., enhanced IFN-dependent degradation of cytosolic DENV RNA.

Nevertheless, we will compare, where possible, model parameter estimates, model structures, and model calibration. For a comparison of model parameter values, see Table S2 below.

***Comparison of the plus-strand RNA virus replication model with our previously published sub-genomic HCV RNA model***

The plus-strand RNA virus replication model is based on cell-infection experiments, modeled as virus entry and vRNA release, while the sub-genomic-HCV RNA model is based on transfected HCV RNA. Therefore, our model and parameter comparison start with the processes involved in HCV RNA translation: the formation of the translation complex $k_{1}$ including the number of ribosomes $Ribo_{tot}$, vRNA translation $k_{2}$, and polyprotein cleavage $k_{c}$. The translation complex formation rate, $k_{1}$, increased from 1 per h per molecule in the sub-genomic HCV RNA model to 1000 mL per molecule per h in the here presented plus-strand RNA virus model. However, in both models, $k_{1}$ was non-identifiable. Interestingly, there is a large difference in the number of ribosomes involved in vRNA translation. While an estimated $Ribo_{tot}$ = 628 molecules are involved in HCV RNA translation in the sub-genomic HCV RNA model, the plus-strand RNA virus replication model estimated only $Ribo_{tot}$ = 0.005 molecules per mL, which was kept constant throughout the model fitting process. Even though the estimated number of ribosomes was identifiable in both models, in the current plus-strand RNA virus replication model, the 95% CI was narrower than in the sub-genomic HCV RNA model. However, the different system units hamper a direct comparison. Furthermore, the number of ribosomes and the translation complex formation rate may be correlated, whereas a faster $k_{1}$ may require fewer ribosomes.

We adapted the HCV RNA translation rate for the current plus-strand RNA virus replication model to account for a full-length HCV genome. The RNA translation rate, $k_{2}$, almost doubled (from $k_{2}=$100 per h to $k_{2}=$180 per h) due to a higher ribosome density, where more RNA-bound ribosomes produce more polyprotein (for more details see S1 Supporting text and Methods). Similar to the increased HCV RNA translation rate, the polyprotein cleavage rate, $k_{c}$, increased and doubled in the plus-strand RNA virus replication model, compared to the sub-genomic HCV RNA model. On the one hand, the polyprotein is produced from a full-length genome that involves genes for non-structural ($P_{N})$ and structural proteins ($P_{S}$), which may increase the polyprotein cleavage rate. On the other hand, in the plus-strand RNA virus model $k_{c}$ was a pan-viral estimate including the fast-replicating CVB3. The 95% confidence interval for $k_{c}$ calculated a lower boundary of 1.18 per h, which lies closer to the estimated $k_{c}$ value from the sub-genomic HCV RNA model but is highly speculative.

Non-structural proteins and vRNA form a replicase complex (RC) with $k_{Pin}$, which differs largely between both models. While the sub-genomic HCV RNA model estimated $k_{Pin}$ = 9.04e-6 per h per molecule, the plus-strand RNA virus replication model estimated $k_{Pin}$ = 4.4 mL per molecule per h. However, both models differed in how the RC formation was modeled. While the sub-genomic HCV RNA model considered host factors that may limit the RC formation rate, the plus-strand RNA virus replication model modeled the RC formation as a saturating process with a maximum RC formation rate and a maximum number of RCs.

For the HCV RNA synthesis, we again adapted its rate ($k_{4}$) to a full-length HCV genome for the plus-strand RNA virus replication model. The HCV RNA synthesis rate decreased (from $k_{4}$ = 1.7 per h to $k_{4}$ = 1.1 per h) due to the longer HCV genome, where we assume that only one polymerase is bound. Following successful minus-strand synthesis, the replication intermediate complex is formed at constant rate $k_{5}$. For $k_{5}$, both models differ again largely in their estimates. The plus-strand RNA virus replication model estimated 6018 mL per molecule per h, while the sub-genomic HCV RNA model estimated 10 per h per molecule. Since this process takes place within the replication organelle, it is not surprising that the replication intermediate complex forms very fast or does not dissociate. Additionally, in the plus-strand RNA virus replication model, $k_{5}$ was fully identifiable, even with a large 95% confidence interval. However, newly synthesized plus-strand HCV RNA may remain within the replication organelle for further synthesis of HCV RNA with constant rate $k_{3}$ or be exported from the replication organelle into the cytoplasm with constant rate $k_{Pout}$. Both processes were faster in the plus-strand RNA virus replication model by several orders of magnitude but fully identifiable. Nevertheless, both processes compete with virus assembly and release in the here presented plus-strand RNA virus replication model. The production and release of infectious virus may need more resources regarding HCV RNA leading to a faster $k_{3}$ and, thus, further HCV RNA synthesis, and HCV protein resulting in a faster $k_{Pout}$ for further HCV RNA translation.

Considering HCV RNA and protein degradation rates, several values for the plus-strand RNA virus replication model have been taken from the sub-genomic HCV RNA model. Nevertheless, for the plus-strand RNA virus replication model, we revised the degradation rate for free cytosolic HCV RNA ($\mu_{RP}$= 0.26 per h), which has been taken from literature and was close to the value estimated in the sub-genomic HCV RNA model ($\mu_{RP}$ = 0.363 per h).

Note that complex formation rates such as $k_{1}$, $k_{3}$, $k_{5}$, and $k_{Pin}$ and molecule numbers and concentrations are difficult to compare due to the difference in the system units—molecules per h versus molecules per mL per h.

***Comparison of the plus-strand RNA virus replication model with our previously published DENV model***

While the coupled DENV model is based on the sub-genomic HCV RNA model, there are slight differences in the model structure between the here presented plus-strand RNA virus replication model and the DENV model, which was coupled to the host cellular immune response. However, both models were developed for a cell-infection experiment and thus include virus entry and DENV release. Nevertheless, in the plus-strand RNA virus replication model, virus attachment and entry from the DENV model have been combined into a single virus entry process. Estimates of virus entry, $k_{e}$, in both models were compatible and identifiable in both models ($k_{e}$ = 0.43 per h versus $k_{e}$ = 0.31 per h). However, the rate of DENV genome release, $k_{f}$, was estimated to be four times slower in the plus-strand RNA virus replication model (0.008 per h) compared to the DENV model (0.031 per h). Those differences in genome release rates may be due to the different infection experiments. While in the DENV model, we fitted $k_{f}$ as a shared model parameter for Huh7 and A549 cell lines, in the plus-strand RNA virus replication model, $k_{f}$ was only fitted to the DENV data measured in Huh7 cells.

Estimated model parameters of processes involved in DENV RNA translation were mostly similar between the plus-strand RNA virus replication model and the DENV model. The translation complex formation rate, $k_{1}$, was estimated at 1000 mL per molecule per h and non-identifiable in both models. We assume that those identifiability issues lie within the model structure since the additional data of HCV and CVB3 did not solve the identifiability issues. However, the total ribosome concentration decreased in the plus-strand RNA virus replication model compared to the DENV model from 2.8 to 0.48 mL per molecule per h. The value for the ribosome concentration estimated in the DENV model may be biased by the ribosome concentration in A549 cells. However, the DENV RNA translation rate was again calculated based on the full-length DENV genome (see Methods) and taken from the DENV model. While in the DENV model, the polyprotein cleavage rate was taken from the HCV model ($k_{c}$ = 1 per h), we estimated $k_{c}$ in the plus-strand RNA virus replication model as a pan-viral parameter as $k_{c}$ = 2.24 per h and, thus, potentially biased by HCV and CVB3.

The formation of the replicase complex, $k_{Pin}$, was four times slower in the DENV model ($k_{Pin}=$0.12 mL per molecule per h) compared to the plus-strand RNA virus replication model ($k_{Pin}=$0.45 mL per molecule per h). However, both models vary in how the replicase complex formation process was modeled, where we included saturation of the formation rate and a maximum number of RCs into the plus-strand RNA virus replication model, while host factors have been excluded. The DENV RNA synthesis rate was taken from the DENV model and calculated from a full-length DENV genome (see S1 Supporting text and Methods). Interestingly, both models estimated the replication intermediate complex formation rate, $k_{5}$, as fast processes ($k_{5}=$1000 versus $k_{5}=$6018 mL per molecule per h). Even though not identifiable in the DENV model, $k_{5}$ was fully identifiable in the here presented plus-strand RNA virus replication mode. A possible explanation for the fast replication intermediate complex formation may be the close proximity of compounds necessary for complex formation within the replication organelle or replicase complex does not disassemble.

Newly synthesized DENV RNA either remains within the replication organelle for further DENV RNA synthesis ($k_{3}$), is transported out of the replication organelle into the cytoplasm for further DENV RNA translation ($k_{Pout}$) or is packaged into virions and released from the cell ($k_{p}$). Being a pan-viral model parameter and, thus, biased by HCV and CVB3, $k_{3}$ was estimated to be more than ten times slower in the plus-strand RNA virus replication model than the DENV model (42 versus 510 mL per molecule per h). Nevertheless, $k_{3}$ estimated with the DENV model (510 mL per molecule per h) lies within the 95% confidence interval of $k_{3}$ estimated with the plus-strand RNA virus replication model (95% CI from 5.5 to 525). Additionally, $k_{3}$ was only identifiable in the plus-strand RNA virus replication model, giving more confidence about the estimated value. The transport from the replication organelle into the cytoplasm, $k_{Pout}$, was a virus-specific model parameter in the plus-strand RNA virus replication model and, thus, directly comparable. While we estimated $k_{Pout}$ as 1000 per h in the DENV model without being identifiable, we were able to refine this estimate for DENV in the plus-strand RNA virus replication model. Here, we estimated an export rate of $k_{Pout}$ = 53 per h which was fully identifiable.

The assembly and release process has been taken from the DENV model for the plus-strand RNA virus replication model. Its parameters for $N_{Ps}$ and $K_{D}$ have been taken from the DENV model and are based on calculations or literature values (see S1 Supporting text and Methods). The virus assembly and release rate, $k_{p}$, increased in the current plus-strand RNA virus replication model by one order of magnitude. However, in the plus-strand RNA virus replication model, $k_{p}$ was estimated pan-viral and is therefore biased by the chronic disease-causing HCV and the fast-replicating CVB3. Furthermore, we consider re-infection in both models, $k_{re}$, which has shown non-identifiable. Due to the high MOI experiment, re-infection may not occur.

Considering degradation rates, we found major differences between the plus-strand RNA virus replication model and the DENV model. Degradation rates for extracellular virus, $\mu_{V}$, and intracellular virus within endosomes, $\mu_{VE}$, have been taken from literature, partially from Zika virus. While $\mu_{VE}$ is similar in both models, the extracellular virus degradation decreased in the here presented plus-strand RNA virus replication model.

Interestingly, the free cytosolic DENV RNA degradation rate, $\mu_{RP}$, was ten times higher in the DENV model compared to the experimentally chosen value for Zika virus. However, both estimates are difficult to compare since $\mu_{RP}$ is a shared parameter in the DENV model and thus may be biased by measurements of A549 cell line. The degradation rate of the translation complex has decreased by two orders of magnitude in the plus-strand RNA virus replication model ($\mu_{TC}$ = 0.001 per h to $\mu_{TC}$ = 0.115 per h), which is now based on the assumption that the translation complex is more stable than free cytosolic RNA, which we directly calculated from $\mu_{RP}$ (see S1 Supporting text and Methods). However, in the DENV model, we added an immune response effect on the translation complex formation, which may affect its degradation in both cell lines. Interestingly, the estimated degradation rate of species within the replication organelle, $\mu_{RO}$, showed a four-times difference between both models. While we took $\mu_{RO}$ for the plus-strand RNA virus replication model from the sub-genomic HCV RNA model, we estimated $\mu_{RO}$ in the DENV model. The four times slower degradation of the replication organelle in the DENV model may be related to the modeled immune response since recognition of DENV RNA depends on the degradation of the replication organelle. However, the degradation of structural and non-structural proteins, $\mu_{P}$, has also been estimated as two orders of magnitude smaller in the DENV model, while we used literature values in the here presented plus-strand RNA virus replication model. Since the degradation rate of viral proteins was largely unidentifiable in the DENV model, the estimate is highly uncertain, which we corrected for literature values in the plus-strand RNA virus replication model.

Table S2: Parameter values and 95% confidence intervals in (). Confidence intervals marked with + hit the set estimation boundary. For references and more details, see Table 2 in main text, Table 1 in [1], and Table S6 in [2].

|  |  | *Mathematical modeling of plus-strand RNA virus replication to identify broad-spectrum antiviral treatment strategies [present study]* | | | | *Replication Vesicles are Load- and Choke-Points in the Hepatitis C Virus Lifecycle [1]* | *A Coupled Mathematical Model of the Intracellular Replication of Dengue Virus and the Host Cell Immune Response to Infection [2]* |
| --- | --- | --- | --- | --- | --- | --- | --- |
| *Parameter* | *Description* | *HCV* | *DENV* |  | *Unit* | *sub-genomic HCV* | *DENV* |
| $\boldsymbol{k}_{\boldsymbol{e}}^{\boldsymbol{i}}$ | Virus entry rate | 10  (1.9, *10^+^*) | 0.31  (0.28, 0.34) |  | 1/h |  | 0.43/h  (0.41, 0.44) |
| $\boldsymbol{k}_{\boldsymbol{f}}^{\boldsymbol{i}}$ | RNA release rate | 10  (1.7, *10^+^*) | 0.008  (0.006, 0.01) |  | 1/h |  | 0.031/h  (0.027, 0.035) |
| $\boldsymbol{k}_{\boldsymbol{1}}$ | Formation rate of the translation complex | 1000  (840, *1000^+^)* | | | mL/molecule /h | 1/h /molecule | 1000 mL/molecule /h  (857,*1000+*) |
| $\boldsymbol{k}_{\boldsymbol{2}}^{\boldsymbol{i}}$ | Virus RNA translation rate | 180 | 100 |  | 1/h | 100/h | 100/h |
| $\boldsymbol{k}_{\boldsymbol{c}}$ | Polyprotein cleavage rate | 2.24  (1.18, 7.4) | | | 1/h | 1/h | 1/h |
| $\boldsymbol{k}_{\boldsymbol{3}}$ | Formation of additional replicase complexes within the replication organelle | 42  (5.5, 525) | | | mL/molecule /h | 10^-4^/h /molecule | 510 mL/molecule /h |
| $\boldsymbol{k}_{\boldsymbol{4}\boldsymbol{m}}^{\boldsymbol{i}}\boldsymbol{=}\boldsymbol{k}_{\boldsymbol{4}\boldsymbol{p}}^{\boldsymbol{i}}$ | Minus- and plus-strand RNA synthesis rate | 1.1 | 1.0 |  | 1/h | 1.7/h | 1.0/h |
| $\boldsymbol{k}_{\boldsymbol{Pin}}^{\boldsymbol{i}}$ | Formation rate of the replicase complex | 4.4  (2.4, 7.5) | 0.45  (0.29, 0.74) |  | mL­­­/molecule /h | 9.04e-6 /h /molecule^2^  (3.85e-7, 2.12e-4) | 0.12 mL­­­/molecule /h  (0.008, 0.016) |
| $\boldsymbol{k}_{\boldsymbol{5}}$ | Formation rate of the replication intermediate complex | 6018  (1549, 68401) | | | mL/molecule /h | 10 /h /molecule | 1000 mL/molecule /h  (748, 1000) |
| $\boldsymbol{k}_{\boldsymbol{Pout}}^{\boldsymbol{i}}$ | Export rate of viral RNA out of the replication organelle | 33  (0.8, 1477) | 53  (16, 432) |  | 1/h | 0.307/h  (0.167, 0.538) | 1000/h  (856.5, 1000) |
| $\boldsymbol{k}_{\boldsymbol{p}}$ | Assembly and release rate | 158  (47, *1000^+^*) | | | mL/molecule /h |  | 11 mL/molecule /h  (0, 486) |
| $\boldsymbol{k}_{\boldsymbol{re}}$ | Reinfection rate | 0.01  (*0.01^+^*, 0.038) | | | 1/h |  | 0.0001/h  (0, 24.4) |
| $\boldsymbol{\mu}_{\boldsymbol{RP}}^{\boldsymbol{i}}$ | Degradation rate of cytosolic viral RNA | 0.26 | 0.23 |  | 1/h | 0.363/h  (0.168, 0.783) | 2.8/h  (1.9, 4.2) |
| $\boldsymbol{\mu}_{\boldsymbol{TC}}^{\boldsymbol{i}}$ | Degradation rate of the translation complex | 0.13 | 0.115 |  | 1/h | 0.181/h  (0.0841, 0.392) | 0.001/h  (0.001, 0.025) |
| $\boldsymbol{\mu}_{\boldsymbol{RO}}$ | Degradation rate of viral RNA and protein within the replication organelle | 0.0842 | | | 1/h | 0.0842/h  (0.0193, 0.366) | 0.028/h  (0.019, +∞) |
| $\boldsymbol{\mu}_{\boldsymbol{P}}^{\boldsymbol{i}}$ | Degradation rate of viral protein | 0.06 | 0.46 |  | 1/h | 0.06/h | 0.001/h  (0.001,0.006)  to 0.0025/h  (0, 0.01) |
| $\boldsymbol{\mu}_{\boldsymbol{L}}$ | Degradation rate of luciferase | 0.35 | | | 1/h | 0.35/h | 0.35/h |
| $\boldsymbol{\mu}_{\boldsymbol{V}}^{\boldsymbol{i}}$ | Degradation rate of extracellular infectious virus | 0.1 | 0.13 |  | 1/h |  | 0.4/h |
| $\boldsymbol{\mu}_{\boldsymbol{VE}}$ | Degradation rate of intracellular virus within the endosome | 0.23 | | | 1/h |  | 0.2/h |
| $\boldsymbol{Rib}\boldsymbol{o}_{\boldsymbol{tot}}^{\boldsymbol{i}}$ | Total ribosome concentration | 0.005  (0.004, 0.007) | 0.48  (0.41, 0.55) |  | molecules/mL | 628 molecules  (68, 5810) | 2.8 molecules/mL  (2.4, 3.3) |
| $\boldsymbol{K}_{\boldsymbol{D}}^{\boldsymbol{i}}$ | Scaling constant for virus | 0.04 | 1.8 |  | virions |  | 1.8 virions |
| $\boldsymbol{N}_{\boldsymbol{P}_{\boldsymbol{S}}}^{\boldsymbol{i}}$ | Number of structural proteins needed to produce 1 virion | 180 | 180 |  | molecules/virion |  | 180 molecules/virion |

1. Binder M, Sulaimanov N, Clausznitzer D, Schulze M, Hüber CM, Lenz SM, et al. Replication vesicles are load- and choke-points in the hepatitis C virus lifecycle. PLoS Pathog. 2013;9: e1003561. doi:10.1371/journal.ppat.1003561

2. Zitzmann C, Schmid B, Ruggieri A, Perelson AS, Binder M, Bartenschlager R, et al. A coupled mathematical model of the intracellular replication of dengue virus and the host cell immune response to infection. Front Microbiol. 2020;11: 725. doi:10.3389/fmicb.2020.00725
