# Supplementary material for: Mathematical modeling of plus-strand RNA virus replication to identify broad-spectrum antiviral treatment strategies
Source: PLoS Comput Biol. 2023 Apr 4;19(4):e1010423. doi: 10.1371/journal.pcbi.1010423 (PMC10104377; doi:10.1371/journal.pcbi.1010423)
Supplement: S1 Table — For simplicity, we assume that both drugs have the same efficacy in combination therapy. The lowest critical drug efficacies to clear the virus-specific infection is highlighted in red (TC = translation complex, RC = replicase complex) (DOCX) [file pcbi.1010423.s005.docx]

| Drug A | Drug B | HCV | DENV | CVB3 |
| --- | --- | --- | --- | --- |
| *TC formation (*$\boldsymbol{k}_{\boldsymbol{1}}$*)* | *-* | 0.96 | 1 | 1 |
| *Translation (*$\boldsymbol{k}_{\boldsymbol{2}}$*)* | *-* | 0.99 | 0.99 | 1 |
| *Polyprotein cleavage (*$\boldsymbol{k}_{\boldsymbol{c}}$*)* | *-* | 0.995 | 1 | 1 |
| *RC formation (*$\boldsymbol{k}_{\boldsymbol{Pin}}$*)* | *-* | 0.99 | 1 | - |
| *RNA synthesis (*$\boldsymbol{k}_{\boldsymbol{4}\boldsymbol{p}}$ *and* $\boldsymbol{k}_{\boldsymbol{4}\boldsymbol{m}}$*)* | *-* | 0.89 | 0.865 | 0.995 |
| *Viral export (*$\boldsymbol{k}_{\boldsymbol{Pout}}$*)* | *-* | 1 | 1 | 1 |
| *Virus assembly and release (*$\boldsymbol{k}_{\boldsymbol{p}}$*)* | *-* | 1 | 1 | 1 |
| *TC formation (*$\boldsymbol{k}_{\boldsymbol{1}}$*)* | ***RNA synthesis (***$\boldsymbol{k}_{\boldsymbol{4}\boldsymbol{p}}$ ***and*** $\boldsymbol{k}_{\boldsymbol{4}\boldsymbol{m}}$***)*** | **0.76** | **0.85** | 0.993 |
| *TC formation (*$\boldsymbol{k}_{\boldsymbol{1}}$*)* | ***RC formation (***$\boldsymbol{k}_{\boldsymbol{Pin}}$***)*** | 0.85 | 0.99 | 1 |
| *Translation (*$\boldsymbol{k}_{\boldsymbol{2}}$*)* | ***RNA synthesis (***$\boldsymbol{k}_{\boldsymbol{4}\boldsymbol{p}}$ ***and*** $\boldsymbol{k}_{\boldsymbol{4}\boldsymbol{m}}$***)*** | 0.90 | **0.85** | 0.99 |
| *Translation (*$\boldsymbol{k}_{\boldsymbol{2}}$*)* | ***RC formation (***$\boldsymbol{k}_{\boldsymbol{Pin}}$***)*** | 0.96 | 0.98 | 0.991 |
| *Polyprotein cleavage (*$\boldsymbol{k}_{\boldsymbol{c}}$*)* | ***RNA synthesis (***$\boldsymbol{k}_{\boldsymbol{4}\boldsymbol{p}}$ ***and*** $\boldsymbol{k}_{\boldsymbol{4}\boldsymbol{m}}$***)*** | 0.90 | 0.87 | **0.98** |
| *Polyprotein cleavage (*$\boldsymbol{k}_{\boldsymbol{c}}$*)* | ***RC formation (***$\boldsymbol{k}_{\boldsymbol{Pin}}$***)*** | 0.997 | 0.999 | 1 |
