# Supplementary figures and images for: Mathematical modeling of plus-strand RNA virus replication to identify broad-spectrum antiviral treatment strategies
Source: PLoS Comput Biol. 2023 Apr 4;19(4):e1010423. doi: 10.1371/journal.pcbi.1010423 (PMC10104377; doi:10.1371/journal.pcbi.1010423)

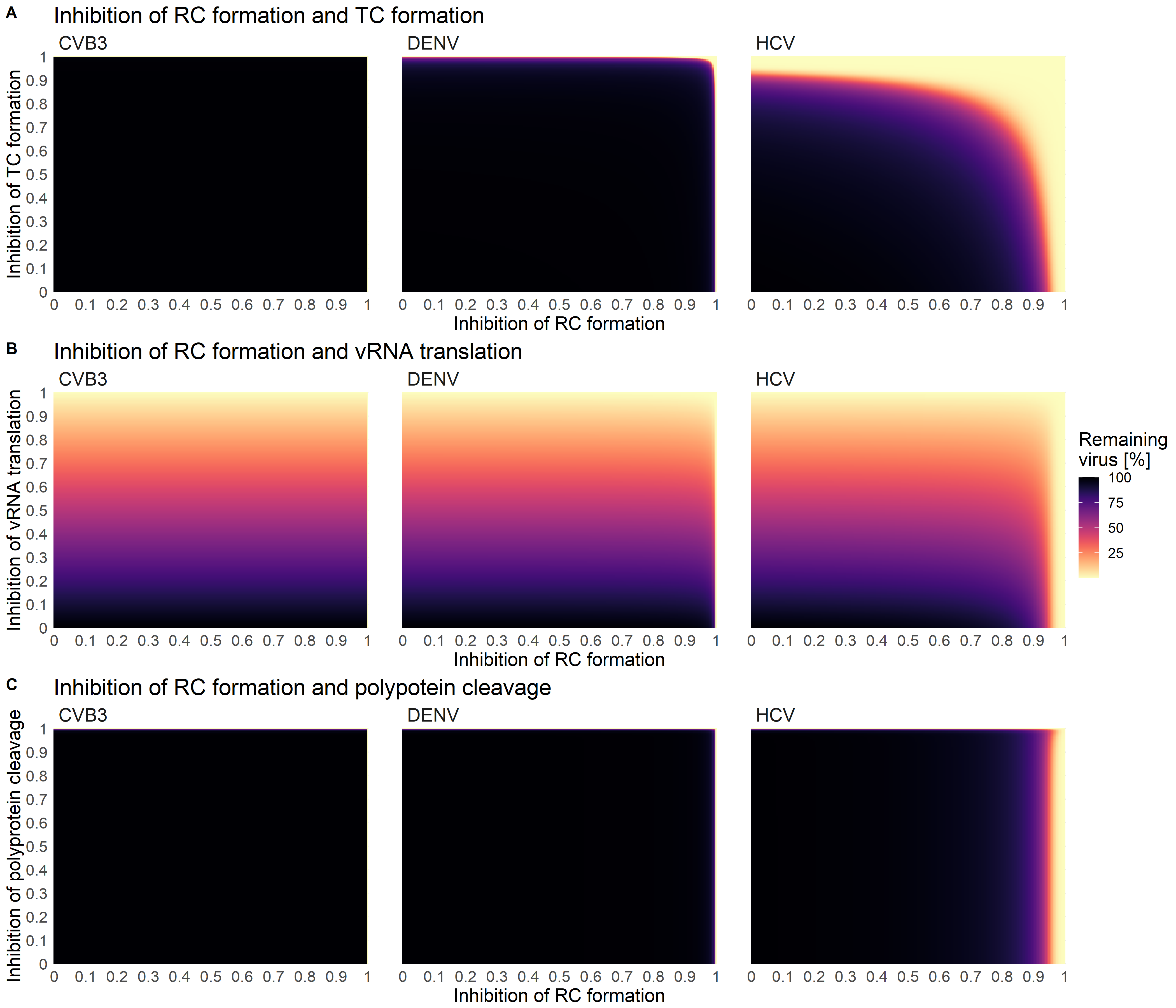

Supplement: S1 Fig — Combined drug effect on A) replicase complex (RC) formation and formation of translation complex (TC), B) replicase complex (RC) formation and polyprotein cleavage, and C) replicase complex (RC) formation and vRNA translation and drug administration in steady state (100 h pi). A successful drug treatment leads to more than 99% viral eradication (light yellow), while an ineffective drug treatment leads to 100% remaining virus (black). (TIFF) [file pcbi.1010423.s006.tiff]

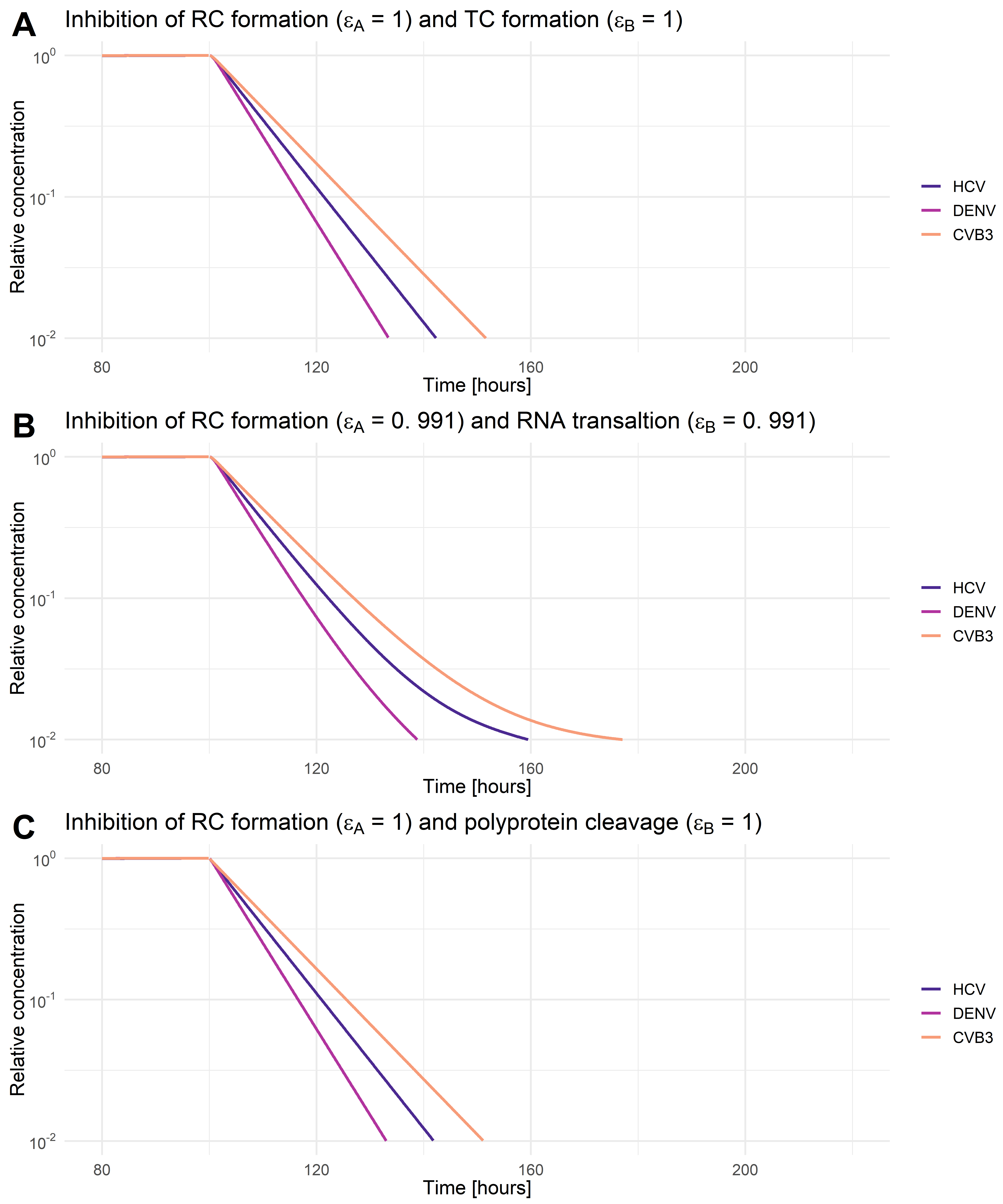

Supplement: S2 Fig — A combined drug effect on A) formation of replicase complex (RC) and formation of translation complex (TC), B) formation of replicase complex (RC) and translation, and C) formation of replicase complex (RC) and polyprotein cleavage. Initiation of treatment was in steady state (100 h pi). The drug efficacy constant (εA and εB) were chosen as minimal efficacies to clear all three viruses. For comparability, virus-specific concentrations in steady state have been normalized to their virus-specific pre-treatment steady-state concentration. A successful drug treatment leads to more than 99% viral eradication (light yellow), while an ineffective drug treatment leads to 100% remaining virus (black) (see S1 Data). (TIFF) [file pcbi.1010423.s007.tiff]
