# Supplementary material for: Mathematical modeling of plus-strand RNA virus replication to identify broad-spectrum antiviral treatment strategies
Source: PLoS Comput Biol. 2023 Apr 4;19(4):e1010423. doi: 10.1371/journal.pcbi.1010423 (PMC10104377; doi:10.1371/journal.pcbi.1010423)
Supplement: Attachment — Submitted filename: response_to_reviewers.pdf [file pcbi.1010423.s008.pdf]

***Reviewer #1:***

**In this manuscript, the authors present findings from both in vitro experiments and mathematical modeling to provide further understanding on plus-strand RNA viruses. They develop a new model, which describes the infection dynamics of HCV, DENV and CVB3. The authors calibrated this model to experimental data and used it to examine the similarities and differences between these virus species that operate on very different time scales. The computational study of intracellular targets for broad-spectrum antivirals will help guide the development of new antivirals for plus-strand RNA viruses. The work is clear and well written. I have a few comments regarding this manuscript.**

***We thank the reviewer for his careful and positive assessment of our work. We have revised the manuscript according to both reviewers suggestions, and hope you will now find the manuscript suitable for publication.***

**Major comments:**

**1. The experiments for HCV, CVB3 and DENV were performed at MOIs of 1, 1 and 10, respectively. In contrast, the estimated initial virus concentrations show values of 0.2, 1 and 1. Do you think this difference affects the results of your experiments and that your simulations correctly reflect the initially available virus material?**

*We do not believe that the initial virus concentration is a crucial parameter in our model. The ability of each virus to attach to the cell surface, enter a cell and release the viral genomic material is more crucial for the infection process in our model, and is the limiting factor. In our simulation experiments, we generated a one-step growth curve without multiple rounds of infection. In this setting, k_re being small is a crucial parameter value. We included this information to the S1 supporting text about parameter assumptions.*
*Please see also the corresponding comment by reviewer two.*

**2. In your model, the total ribosome concentration (Ribo_tot) appears to be the most crucial factor influencing the infection dynamics and time scales for the three viruses. Related to this I have four questions:**

**(a) You note that this total ribosome concentration is only a fraction of the total number of ribosomes per cell specific to each virus. Does this number represent the ability of ribosomes to bind to these viral genomes and the higher and lower values represent some kind of binding affinity or overall availability of these ribosomes?**

*The ribosome concentration modeled here represents only the fraction involved in viral translation. We did not consider the binding affinity; however, we did consider the ribosome density (see S1 supporting text).*

**(b) It seems like this total ribosome concentration induces the large differences in infection time scales in your model (together with the high synthesis rate for CVB3). How reliable would this deduction be and was this already considered previously?**

*We tested our model for different scenarios and found that the number of ribosomes available for vRNA translation seems more crucial than the vRNA synthesis rate. To our knowledge, this has not been considered before.*

**(c) You derive conclusions regarding the suppression of host cell mRNA translation from the model. However, such a mechanism is not implemented in the model itself. Do you base this solely on the values estimated for total ribosome concentration or is there further supporting evidence from your model? Moreover, is this suppression capability the major factor for the ribosome concentration and ultimately induces the different time scales of the viruses?**

*You are correct, we did not attempt to model host cell mRNA translation as that is well outside the scope of this model. Ribosomes represent a crucial host factor in our model. We conclude that the number of ribosomes available for viral RNA translation is crucial for replication efficiency and host shut-off. Hence, the more ribosomes a virus uses (hijacks) for its replication, the fewer ribosomes are available for cellular mRNA translation. Also, we show that increasing the number of ribosomes involved in vRNA translation increased vRNA levels by 2 orders of magnitude, which is substantial. We also speculate that the more viral RNA, the more virus may be produced, hence, the more severe an infection.*

**(d) Why is the ribosome concentration included as the only host factor in the model, was it previously identified as the most crucial factor? Furthermore, why is the actual concentration of ribosomes included and its effect is not represented only in the reaction rates (TC formation and protein translation)?**

*Every virus uses different host factors. Identifying host factors that a whole group of viruses uses for their replication represents an important challenge for the future, and has among others been attempted by us in the context of the SysVirDrug projected funded by the European Union using RNAi screening data - with limited success. However, all our studied viruses need ribosomes for vRNA translation. The virus may hijack other host factors, but either it is not 100% clear what these host factors are doing, or they are virus-specific. Therefore, we included ribosomes as the only explicit host factor in our model. Other host factors are implicitly included in reaction constants,but are not explicitly included in the model.*
*We furthermore kept the ribosome concentration constant, because we do not believe that the cell may be limited by ribosomes in general. However, we do believe that the number of ribosomes available for vRNA replication may be important in the vRNA replication efficiency, as shown by increasing or decreasing its number. Interestingly, varying only this single model parameter led to an increase or decrease in vRNA by 2 to 3 orders of magnitude. We could not achieve the same result by increasing or decreasing the vRNA synthesis rate. In our model,*

*the ribosome concentration only affects the rate of TC formation and protein translation, as we are unaware of other steps in our model where ribosomes should be included.*

**3. The entry and RNA release rates (k_e and k_f) for the three viruses show large differences. HCV rates are deemed non-identifiable and are estimated to the upper boundary, while the other two have significantly lower rates. These rates lead to the large majority of initially provided virus material of HCV to be available in the cytoplasm after around 0.3 h. However, based on the estimated rates, only a marginal fraction of the infecting viruses for DENV and CVB3 actually reach the cytoplasm (after 10 h, only around 5 and 12% of the total initial virus material, respectively). For these two viruses, this small amount of provided genomic material is amplified via high translation and replication rates. Ultimately, only a fraction of the infecting virus is used for replication and everything released from endosomes after the first minutes/hours is irrelevant. Thus, these low rates, especially for k_f, seem quite unrealistic to me. Can you comment on that?**

*Since the parameters describing the rates of HCV entry and RNA release (k_e and k_f) are non-identifiable, we cannot comment on these rates. However, for DENV and CVB3, those processes may be rate-limiting steps in the life cycles of these viruses. Since we carried out our measurements in Huh7 cells for all viruses, we assume both processes are slow because the studied viruses usually do not infect hepatocytes. They can be infected but most probably not as efficiently as cells in a natural infection. Cell entry and, thus, the ability to infect a cell always represent a barrier to infection.*
*We added that information to the discussion; see virus-specific differences/ virus internalization.*

**4. In line 315, it is mentioned that the newly formed double-stranded RNA and the non-structural proteins are released from the RO. However, that is not depicted in the model scheme and the following steps appear to still occur inside of the RO. Do the double-stranded RNA and the non-structural proteins actually leave and re-enter the RO?**

*We thank the reviewer for carefully reading the manuscript. Indeed, the non-structural proteins are released from the replicase complex, the RC. We changed the sentence accordingly.*

Minor comments:

1. Short title: plos-strand --> plus-strand
*done*

2. Line 77-78: significant burden --> significant burdens
*done*

3. 137: RNA translation[22] --> RNA translation [22]

*done*

4. 188: prof. R. --> Prof. R.
*done*

5. 537: Splitting of two words over the whole line.
*done*

6. 552: The abbreviation “IRES“ is introduced in line 690, but already used in lines 552-553.
*done*

7. 600: Table,) --> Table)
*done*

8. 697: shup down --> shut down
*done*

*We thank the reviewer for catching all of these types.*

**9. Figure 1: In the model scheme, the replicase complex inside the replication organelle is shown as RC^RO. However, in the model equations it is used as only RC while the other variables have the exact same names as in the model scheme. An adjustment of the denomination either in the scheme or in the equations would increase clarity.**

*We thank the reviewer for catching this. The scheme has been changed.*

**10. Figure 7, 8, S1: On the x-axis, it seems like 0.9 is followed by 10. If possible, separate the figures a bit further to split up the numbers 1 and 0.**

*We agree that the x axis may be misleading. We separated the figures.*

**11. You have determined which processes are virus-specific by employing the AIC and considering parameter identifiability. Do you think by using a larger set of experimental data to inform the model additional distinctions between pan-viral and virus-specific processes could be made?**

*We agree that more data would have given more confidence in the model calibration. However, the dynamics of various model species, such as translation and replication complexes, are challenging to measure. Additionally, measuring the dynamics in different compartments is also challenging.*

**12. The experimental data and models were not available at the time of the review and will apparently be made available later. Thus, the results could not be reproduced.**

**Please supply all code, well documented and with instructions, such that a reader could rerun your simulations and reproduce your figures/tables/results.**

*A github link to model and data has been added.*

***Reviewer #2:***

**This work aims to develop a generic mathematical model for plus-strand RNA viral replication to identifying antiviral treatment strategies. To do so, the authors modified/simplified their previous published models into the proposed generic model that was calibrated against previous published viral kinetic experimental data of hepatitis C virus (HCV), dengue virus (DENV), or coxsackievirus B3 (CVB3) in cell cultures. The authors suggest, via model selection process against the measured experimental data, that many viral-host parameter were similar among the 3 investigated viruses. Thereafter, to examine antiviral treatment strategies they simulated antiviral perturbations in each virus-specific model at the beginning of infection (time 0) and 100 hr post infection, ie., at steady state. Comments and suggestions follow**

*We thank the reviewer for carefully reading our manuscript and for the insightful comments. We have addressed all of the mentioned points, and hope the reviewer will now find the manuscript suitable for publication.*

**Experimental data:**
**a. It would be important to be clear how the data were obtained (digitized from a figure, performed by the authors, or had access to the raw data)**

*The data was obtained as raw data. The DENV data has been used before, but we have access to the raw data. We added a sentence to the manuscript (method section) for clarification.*

**b. The 3 investigated viruses have different cell tropism (as summarized in Table 1), with DENV not infecting hepatocytes et all so it would be important to explain why Huh7 cells were used. Also, for HCV experiments Lunet-CD81 cells were used so it is not clear whether they are Huh7 cells.**

*HCV is a challenging virus to replicate in vitro. The Huh7 cell line is established and the only cell line that allows studying HCV replication to date. Using the same cell line for all viruses, thus Huh7 cells, allowed us to study the life cycles of the three viruses without the perturbation of different subsets of host factors and, more importantly, without an intrinsic immune response. We added that information to the discussion/virus-specific differences.*

*Huh7 Lunet-CD81 cells are Huh7-derived and support a comparable vRNA replication. The additional expression of CD81 on the cell surface enhanced viral spread, which is otherwise impaired in Huh7-Lunet cells. For more details, see reference 49.*

**c. DENV experiment was used MOI=10 while in the others it was MOI=1. Is that was accounted in the in silico modeling?**

*We did not account for the MOI for the initial virus concentration due to the different cell tropisms of the three viruses. Since it is unclear how many viruses bind to the cell surface, we estimated the initial virus concentration. However, we did account for multiple rounds of infection with the k_re model parameter that occurs with low MOI. However, re-infection or multiple rounds of infection did not occur in our model, and the model parameter k_re was non-identifiable. However, information about initial virus concentration to the S1 supporting text about parameter assumptions.*

**d. How many cells were placed in each well in each experiment? I wonder whether the units FFU/ml and molecules/ml might have differed if the number of cells in each well is different.**

*The raw data was normalized in order to account for a consistent model unit which was copies/ml/cell or PFU/ml/cell.*

**Modeling design/approach**
**While the model selection processes are impressive and provide a decent agreement between the experimental data and the mathematical models, the paper is not an easy to digest where 3 different viruses with different outcomes (acute vs chronic) and cell tropism are compared and discussed. Since models were already developed for HCV by the authors and there are already approved potent drugs for HCV cure, it seems that the generic model could be presented first in the context of HCV. E.g., showing how the generic model agrees with the previously more complex published HCV model, then show HCV-drug perturbation simulations, and if possible, add some experimental data under anti-HCV that should be available in the literature or from the labs of the co-authors of the current paper.**
**For DNEV, the recent modeling paper that was developed by the authors could be compared with the generic model, at first, bringing confidence. Explain how a virus that does not infect hepatocytes can be investigated in Huh7 cells. Then discuss relevant drug perturbations for DENV and explain why perform these simulations at steady state for a virus that is spontaneously cleared (2-3 weeks infection duration as noted in Table 1).**
**For CBV3 it would be important for the readers to know which in silico models have been developed (or not) thus far. How the generic model behaves in a very short time (~10 hr of experiment, Fig. 2C) before death of infected cells occurred (which parameters are different compared to HCV and DENV)? How the generic model can be useful if it reaches a steady state contrary to the experimental data (at least beyond 10 hr)? How relevant are the simulations of VBV3 drug perturbations at steady state if the**

**experimental sys does not reach s.t.? These questions are highly important to convince the readers about the overall ambitious approach of the study to develop such a generic model and broadly test antivirals before and after steady state has reached (in the in silico model).**

*We thank the reviewer for the suggestions, and we considered different designs and approaches. However, we concluded to focus on similarities and virus-specific differences in the plus-strand RNA virus life cycle rather than discussing individual models for each virus. When we fit the three different viruses simultaneously, we changed the model structure slightly for each of the viruses, where we simplified (virus entry) or extended processes (carrying capacity of replicase complexes) and excluded host factors (except ribosomes) in cases where the model processes may not be comparable for the three viruses.*

*Furthermore, comparing our current HCV predictions with previously published clinical data may be biased because the model uses different virus strains and a cell line rather than in vivo human hepatocytes. In our previous HCV modeling study, we used a sub-genomic replicon without structural proteins and, thus, without virus production. In addition, our previous DENV publication took the immune response into account. We compared the DENV life cycle in the absence and presence of the immune response.*

*However, we describe and discuss the five key virus-specific differences and their parameter values, such as virus internalization, viral genome release, viral RNA translation, formation of the replicase complex, and viral RNA export from the RO into the cytoplasm.*

*Drug perturbations in a steady state ensure we account for steady virus replication and production. Thus, the steady states represent the optimum for the virus, where an earlier drug perturbation may bias the in-silico drug analysis toward possible non-established replication machinery. However, it is important to see how the sensitivity changes during infection and which processes may represent the most likely potent drug targets throughout the infection. Since we performed our experimental measurements in Huh7 cells and thus in the absence of an intrinsic immune response, there is no viral clearance. Thus we only included natural degradation processes in our model.*

**Minor questions/comments**

**1. Why the in-silico HCV "Virus" curve starts so low (Fig. 2A) compared to DENV and CBV3 shown in figures 2B and 2C.**

*For HCV, infectivity titer measurements start 20 hours post-infection. Since we did not have earlier measurements where we could see virus uptake as in DENV, we only modeled the release of the virus from the cell. Thus, the predicted virus concentration only represents virus production.*

*The figure caption (Fig 2) was incorrect. We corrected the figure captions in Fig 2. For more information, see S1 supporting text.*

**2. Why to increase number of ribosomes (lines 964-7)?**

*Ribosomes represent the only host factor in our model, but their virus-specific concentration varied by several orders of magnitude. We increased or decreased the number of ribosomes since it has been shown that the three viruses we studied pursue different strategies to shut off or shut down the host's mRNA translation (see Table 1). In addition, varying only the number of ribosomes was enough to increase or decrease the amount of synthesized vRNA by 2 to 3 orders of magnitude. We also tested the viral RNA synthesis rate for a comparable increase (or decrease) in vRNA but could not achieve the same result.*

**3. About 30 of model parameter values are not shown (line 351). Would be nice to add a Table for that and the source for the values.**

*You can find those values in Table 2 marked with *. Details about their calculation and other model parameter assumptions are described in S1 supporting text.*

**4. Fig. 2: Consider to use different model curves and symbols that could be easily seen using B&W printers.**

*We changed the Fig 2 and the figures in the supporting material accordingly.*

**Suggested study limitations to acknowledge:**

**1. The model does not account for drug resistance which was a big challenge to overcome in developing antivirals against HCV.**

*We agree that drug resistance represents a big challenge in developing and using antivirals. However, we aimed to identify common processes that may be sensitive to possible antiviral treatment. The mechanism of drug resistance was outside of the scope of this study.*

**2. Different labs using different viruses may have significant effect on the results.**

*We agree with the reviewer that our model predictions are not universal. Different cell lines and different viruses may lead to different results. More research is needed to understand cell-line and virus-specific replication. However, our current study shows that it is important to study several members of the same virus group and may represent an initial step towards developing broad-spectrum antivirals - a big challenge for the future. Please see limitation 2 and 3 in the discussion.*
