# Supplementary material for: Mathematical modeling of plus-strand RNA virus replication to identify broad-spectrum antiviral treatment strategies
Source: PLoS Comput Biol. 2023 Apr 4;19(4):e1010423. doi: 10.1371/journal.pcbi.1010423 (PMC10104377; doi:10.1371/journal.pcbi.1010423)
Supplement: Attachment — Submitted filename: response-to-reviewer.pdf [file pcbi.1010423.s009.pdf]

Response to Reviewer

Reviewer #2: While some concerns have been addressed, the major ones - pertaining modeling design/approach - were not. Here are the comments that have not been addressed in the revised manuscript.

We apologize for not addressing the reviewer's comments and thank the reviewer for the insightful comments and the opportunity to further improve our manuscript.

While the model selection processes are impressive and provide a decent agreement between the experimental data and the mathematical models, the paper is not easy to digest where 3 different viruses with different outcomes (acute vs chronic) and cell tropism are compared and discussed. Since models were already developed for HCV by the authors and there are already approved potent drugs for HCV cure, it seems that the generic model could be presented first in the context of HCV. E.g., showing how the generic model agrees with the previously more complex published HCV model, then show HCV-drug perturbation simulations, and if possible, add some experimental data under anti-HCV that should be available in the literature or from the labs of the coauthors of the current paper.

We appreciate the reviewer's suggestion about changing the order of Results and/or Discussion by discussing the three viruses one after the other. However, discussing the single viruses one by one was not the aim of this study. Our manuscript's scope was comparing three different (but related) viruses by identifying and discussing pan-viral or virus-specific processes.

Nevertheless, for interested readers, we added a detailed comparison of our here presented plus-strand RNA model with our previously published HCV model (and DENV, see below) as supporting material (see S3 Supporting text). We have decided to add this material as supporting text, and not in the main manuscript, due to its extent.

We would furthermore like to point out that the comparison of the two models is not as straightforward as it may seem at first sight, for several reasons:

Both models have a different system units: While the here presented plus-strand RNA virus model has the system unit molecules per mL per hour per cell, our previous HCV model has the system unit molecules per hour per cell. However, we compared the model parameters of both studies where possible but also gave explanations where the comparison may be difficult.

The virus strains are different: In our previously published HCV model, we studied the replication of sub-genomic RNA, which does not contain structural proteins. In the here presented study, we modeled the life cycle of full-length HCV RNA, which can infect cells and produce viruses. Due to the sub-genomic HCV RNA, we transfected cells with the viral genome, while in the here presented study, we infected cells with the virus. Therefore, the different study designs may make a comparison biased. A longer RNA genome may lead to different rate constants, which we re-calculated and explained. See S1 supporting

text for details about calculation, further explanations, and references to fixed parameter assumptions. However, all estimated model parameters may be affected by different genome lengths.

Both models were developed for a different purpose: In our previously published HCV model, high- and low-permissive cells were compared, thus, cell-line differences. In the here presented study, we compared different viruses. Therefore, comparing the estimated model parameters between both models may be biased, where most of the estimated model parameters in the study here are pan-viral and, thus, summarize three different viruses. In the case of pan-viral processes, we used the 95% confidence bands, which may reflect virus-specific differences.

We did not compare our drug perturbation simulations with in vitro experiments but with real in vivo drug recommendations (see Direct-acting antivirals against HCV in section Broad-spectrum antivirals? of the Discussion). Explaining and validating our in silico drug simulations by a real-world application seemed more appropriate for our scope.

For DENV, the recent modeling paper that was developed by the authors could be compared with the generic model, at first, bringing confidence. Explain how a virus that does not infect hepatocytes can be investigated in Huh7 cells. Then discuss relevant drug perturbations for DENV and explain why performing these simulations at steady state for a virus that is spontaneously cleared (2-3 weeks infection duration as noted in Table 1).

As with the previously published HCV model, we compared the presented plus-strand RNA model in detail with the previously published DENV model in S3 Supporting text. Since we used the experimental measurements from our previous DENV model for the here presented model and having the same system unit for both models, comparing estimated model parameters gave interesting insights about the model structure in general, such as model identifiability. Nevertheless, both models have differences in their design based on their purpose, such as the presence of the immune response that may bias parameter estimation and, again, pan-viral model parameters that have been estimated for three viruses. However, we used 95% confidence bands of those parameters where necessary.

Even though hepatocytes do not support the natural DENV infection, DENV RNA was isolated from different organs and tissues, including the liver. However, whether DENV replicates in the liver is under debate [see references in the manuscript]. The choice of the Huh7 cell line for the DENV experiments was based on our aim to standardize the experiments as much as possible. The establishment of a cell line that supports viral replication is a challenging task. However, the Huh7 cell line has been shown to support the replication of several viruses, such as DENV, chikungunya, Zika, poliovirus, SARS-CoV-2, and other viruses [see references in manuscript]. Additionally, due to its defective RIG-I signaling, the Huh7 cell line has the advantage of studying viral replication without perturbances by the cell's immune response.

Furthermore, the standardized experimental design has another advantage of mostly host-factor equality. Different cell lines may have different host factors highjacked by the different viruses and used for viral replication. We have shown that in our previous DENV model, that host factors in the A549 cell line surprisingly supported DENV replication even in the presence of the cellular immune response (interferon and ISGs). We added some clarification to the manuscript and explained the choice of the cell line.

Simulating in silico drug effects in steady state has the advantage of studying the system in the equilibrium of vRNA replication/virus production and vRNA degradation/virus clearance and, thus, an established infection. Therefore, we can ignore a potential bias of the drug effect when the vRNA translation and replication machinery has to be established or host cellular and viral resources are exhausted, leading to inefficient viral RNA replication and, thus, virus production. Thus, the steady state represents the optimum of vRNA replication but also degradation to maintain virus production. However, we were also interested in studying a potential drug effect early in infection that may prevent the establishment of the vRNA replication machinery. Therefore, studying a drug effect in a steady state or early in infection may give us an idea of which steps in the viral life cycle must be perturbed for treatment or prevention. For clarification, we added this explanation to the manuscript.

No direct-acting antiviral drugs exist for dengue, and no therapy has been approved. We added a sentence for clarification to the manuscript.

For CBV3 it would be important for the readers to know which in silico models have been developed (or not) thus far. How the generic model behaves in a very short time (~10 hr of experiment, Fig. 2C) before death of infected cells occurred (which parameters are different compared to HCV and DENV)? How the generic model can be useful if it reaches a steady state contrary to the experimental data (at least beyond 10 hr)? How relevant are the simulations of VBV3 drug perturbations at steady state if the experimental sys does not reach s.t.? These questions are highly important to convince the readers about the overall ambitious approach of the study to develop such a generic model and broadly test antivirals before and after steady state has reached.

There are only few studies modeling CVB3, and hardly any focusing on the intracellular replication. Nevertheless, we found and added information about a CVB3 model, which includes the immune response.

A comparison of HCV, DENV, and CVB3 regarding their replication efficiency and, thus, identifying pan-viral and virus-specific processes were the scope of the manuscript, which we explained throughout the manuscript in detail.

CVB3 is a lytic virus that replicates fast but also damages the infected cell. Even though the experimental measurements do not reach a steady state, our model can help study CVB3 replication in a steady state. The advantages of a steady state are explained above in the DENV paragraph, and we added some additional explanations to the manuscript.

We furthermore, we added information about the lack of direct-acting antiviral drugs for CVB3.
